# Model-based comorbidity clusters in patients with heart failure: association with clinical outcomes and healthcare utilization

**DOI:** 10.1186/s12916-020-01881-7

**Published:** 2021-01-18

**Authors:** Claudia Gulea, Rosita Zakeri, Jennifer K. Quint

**Affiliations:** 1 Department of Population Health, National Heart and Lung Institute, Imperial College London, London, UK; 2 NIHR Imperial Biomedical Research Centre, London, UK; 3 British Heart Foundation Centre for Research Excellence, King’s College London, London, UK; 4 Royal Brompton & Harefield NHS Foundation Trust, London, UK

**Keywords:** Comorbidity, Hospitalization, Mortality, Resource use

## Abstract

**Background:**

Comorbidities affect outcomes in heart failure (HF), but are not reflected in current HF classification. The aim of this study is to characterize HF groups that account for higher-order interactions between comorbidities and to investigate the association between comorbidity groups and outcomes.

**Methods:**

Latent class analysis (LCA) was performed on 12 comorbidities from patients with HF identified from administrative claims data in the USA (OptumLabs Data Warehouse®) between 2008 and 2018. Associations with admission to hospital and mortality were assessed with Cox regression. Negative binomial regression was used to examine rates of healthcare use.

**Results:**

In a population of 318,384 individuals, we identified five comorbidity clusters, named according to their dominant features: low-burden, metabolic-vascular, anemic, ischemic, and metabolic. Compared to the low-burden group (minimal comorbidities), patients in the metabolic-vascular group (exhibiting a pattern of diabetes, obesity, and vascular disease) had the worst prognosis for admission (HR 2.21, 95% CI 2.17–2.25) and death (HR 1.87, 95% CI 1.74–2.01), followed by the ischemic, anemic, and metabolic groups. The anemic group experienced an intermediate risk of admission (HR 1.49, 95% CI 1.44–1.54) and death (HR 1.46, 95% CI 1.30–1.64). Healthcare use also varied: the anemic group had the highest rate of outpatient visits, compared to the low-burden group (IRR 2.11, 95% CI 2.06–2.16); the metabolic-vascular and ischemic groups had the highest rate of admissions (IRR 2.11, 95% CI 2.08–2.15, and 2.11, 95% CI 2.07–2.15) and healthcare costs.

**Conclusions:**

These data demonstrate the feasibility of using LCA to classify HF based on comorbidities alone and should encourage investigation of multidimensional approaches in comorbidity management to reduce admission and mortality risk among patients with HF.

**Supplementary Information:**

The online version contains supplementary material available at 10.1186/s12916-020-01881-7.

## Background

Heart failure (HF) is currently classified using ejection fraction (EF) [1, 2]. There is increasing recognition that this does not relay the full picture of HF as a complex and heterogeneous syndrome, including both cardiovascular and non-cardiovascular factors implicated in its pathophysiology and prognosis [3–8].

Previous studies have investigated distinct subgroups of HF, but most had strict inclusion criteria (i.e., only hospitalized [9, 10] or registry cohorts [11], heart failure with preserved (HFpEF) [12, 13] or reduced (HFrEF) ejection fraction [14], patients enrolled in randomized controlled trials [RCTs]) [14–17], or included patients from specific geographical areas [11], and thus are not representative of the general Western HF population. While clinical characteristics related to cardiac structure and function were generally available in such studies, allowing for detailed characterization, these data are frequently not available in population studies. This limits the possibility of replication across larger cohorts from administrative databases, where such variables are not recorded, as well as the generalizability of identified subgroups in a routine clinical setting.

As comorbidities are frequent in HF and affect both outcomes and treatment of patients, there may be opportunities to better characterize this population, using routinely collected data. By including a large number of patients (inpatients and outpatients) identified over a period of 10 years in the United States (US) using medical claims data, we aimed to capture and describe comorbidity clusters in patients with HF, using a model-based approach. We also compared clinical outcomes (admission to hospital and mortality) and prescriptions for guideline-recommended pharmacological treatments and healthcare resource used. We hypothesized that there will be significant differences in both clinical and utilization outcomes between clusters as well as differential prescription rates of HF medication.

## Methods

We conducted a retrospective analysis using the OptumLabs Data Warehouse® (OLDW) [18], which contains longitudinal health information on over 100 million commercial enrollees representing a diverse mixture of ages, ethnicities, and geographies across the US, including all 50 states. The administrative claims data in OLDW includes medical, pharmacy claims, and laboratory results for commercial and Medicare Advantage with part D prescription drug coverage patients. The study was exempt from institutional review as it included de-identified data. We identified all individuals at least 18 years old with incident HF, defined as having at least one episode of acute HF that resulted in hospital admission within the study period (January 1, 2008, to January 1, 2019) or at least two outpatient claims on different dates within the study period, with any International Classification of Diseases, Ninth or Tenth Revision [ICD9, ICD10] HF code in any position on the claim. In order to ensure that patients had incident HF, we required them to have at least 12 months of continuous medical coverage with no claim for a HF diagnosis before inclusion (baseline period) and 12 months follow-up thereafter. The earliest claim was used as the index HF diagnosis date when patients were identified using outpatient claims alone; alternatively, the index date was the date of admission to hospital for hospitalized patients. Patients with rheumatic HF (ICD9 code 39891, ICD10 code I09.81) were excluded as the cause for this type of HF is specifically rheumatic fever, which is infectious and less likely related to comorbidity burden, in contrast to other causes of HF. Further details are available in Additional file 1: Supplemental Methods and Table S1.

Comorbidities included other cardiovascular conditions (atrial fibrillation [AF], coronary artery disease [CAD], peripheral artery disease [PAD], cerebrovascular accident [CVA], and hypertension), metabolic conditions (diabetes mellitus, obesity), mental health (depression, alcohol misuse disorder), neurological (dementia), cancer, peptic ulcer, liver disease, renal failure, anemia, and chronic obstructive pulmonary disease [COPD]. These were identified using ICD9 and ICD10 codes recorded any time before the diagnosis of HF (obesity and anemia were assessed in the previous 12 months only as they are potentially transient conditions).

We captured pharmacy prescription claims for the following: cardioselective and non-cardioselective beta-blockers, angiotensin-converting-enzyme inhibitors [ACEis] or angiotensin receptor blockers [ARBs], mineralocorticoid receptor antagonists [MRA], thiazide, potassium sparing, and loop diuretics (Additional file 1: Table S2).

Our main outcome was all-cause hospitalization, defined as the first non-elective admission with at least one overnight stay, occurring within 1 year of, but not including the date of HF diagnosis. Secondary outcomes included mortality, HF-specific hospitalization, in- and outpatient healthcare resource use, and costs.

### Statistical analysis

Latent class analysis (LCA) [19] was conducted using 12 comorbidity variables: AF, anemia, CAD, cancer, COPD, CVA, diabetes mellitus, depression, liver disease, obesity, PAD, and renal failure (Additional file 1: Supplemental Methods – Latent Class Analysis [10, 11, 15]). Maximum-likelihood estimation was used to identify clusters for a range of 2 to 9 groups, and a 5-class model was chosen (Additional file 1: Figure S1-S2, Table S3). Patients were assigned to each cluster according to their highest probability of membership to the group. Uncertainty in class membership (Additional file 1: Table S4) was explored (Additional file 1: Sensitivity analysis [20, 21]). The differences in baseline characteristics between comorbidity clusters were presented using chi-squared and Kruskall-Wallis tests as appropriate. We corrected for multiple testing in the tables using the Bonferroni correction and carried out post hoc Dunn tests to assess differences.

Admission to hospital and mortality were analyzed using Cox proportional-hazard regression models to calculate hazard ratios (HRs) and 95% confidence intervals (CIs). Univariate Kaplan-Meier curves for admission to hospital are shown stratified per cluster, with differences between groups tested using the log-rank test and adjusted for multiple testing using the Bonferroni correction. For admission analysis, patients were followed up for 12 months after receiving a HF diagnosis or censored at disenrollment or death. For mortality, patients were followed up to a censoring date of 1 January 2019, or at disenrollment, whichever came first. This resulted in a maximum follow-up time of 120 months (median and IQR, 30 months, 18–51 months). We assessed the proportional hazards assumption using Schoenfeld residual plots [22]. Where this assumption was not met, outcomes were modeled using time-dependent coefficients [23]. All models were adjusted for baseline characteristics: age, sex, race, education, medical insurance status, place of diagnosis (in- or outpatient), HF medications, and comorbidities not used in the clustering step—hypertension, dementia, peptic ulcer, and alcohol misuse disorder. Incidence of death was calculated as the number of patients who died divided by the total person-months. Negative binomial regression models were used to assess the association between comorbidity clusters and the rate of outpatient, office and emergency room visits, long-term stays, inpatient admissions, and length of stay during 1-year follow-up. Rate ratios and 95% CI were calculated, while adjusting for confounders as mentioned above. For a subset of patients with available data, we conducted additional analyses adjusting for EF and smoking status and tested for interaction between cluster and EF. Additionally, we explored the association between the main outcome and the absolute number of comorbidities. All tests were performed 2-sided. Statistical analyses were performed using R v3.6.2 [24].

## Results

### Baseline characteristics

We identified 318,384 patients with incident HF between January 1, 2008, and January 1, 2018. Baseline characteristics are presented in Table 1. The median age was 73 years (inter-quartile range 63–80) and 51.4% were female. Hypertension (95.2%) was the most common comorbidity, followed by CAD (67.7%), PAD (44.5%), and diabetes (43.7%). The majority of patients were high-school educated or above, 69.5% were White, 14.5% were Black, and less than 20% were Hispanic or Asian. Prescriptions of guideline-directed HF medication at HF diagnosis were relatively low: 46.7% of patients were prescribed ACEis/ARBs, 38.4% cardioselective beta-blockers, and 15% non-selective beta-blockers. The low-burden cluster was comprised almost entirely of patients with less than five comorbidities (93.4% of all patients in this group), while fewer patients in the anemic (48.9%) and metabolic groups (40.7%) had less than five comorbidities. Conversely, the overall burden of comorbidity was greater in the metabolic-vascular and ischemic clusters with almost all patients in the metabolic-vascular group (98%) and a majority in the ischemic group (84. 5%) having five or more comorbidities.

**Table 1:** Baseline characteristics per comorbidity cluster

	Low-burden (*n* = 83,577)	Metabolic-vascular (*n* = 73,284)	Ischemic (*n* = 83,283)	Anemia (*n* = 14,959)	Metabolic (*n* = 63,281)	Overall (*n* = 318,384)
**Age**						
Median [IQR]	71 [60, 79]	73 [66, 80]	78 [70, 82]	73 [62, 81]	67 [57, 74]	73 [63, 80]
**Sex**						
Female	42,440 (50.8%)	36,716 (50.1%)	40,818 (49.0%)	9529 (63.7%)	34,017 (53.8%)	163,520 (51.4%)
Male	41,137 (49.2%)	36,568 (49.9%)	42,465 (51.0%)	5430 (36.3%)	29,264 (46.2%)	154,864 (48.6%)
**Comorbidities**						
AF	26,090 (31.2%)	35,031 (47.8%)	44,233 (53.1%)	1968 (13.2%)	16,844 (26.6%)	124,166 (39.0%)
CAD	43,999 (52.6%)	63,890 (87.2%)	73,417 (88.2%)	1771 (11.8%)	32,617 (51.5%)	215,694 (67.7%)
CVA	15,165 (18.1%)	44,157 (60.3%)	58,088 (69.7%)	3691 (24.7%)	8314 (13.1%)	129,415 (40.6%)
PAD	10,766 (12.9%)	56,137 (76.6%)	64,058 (76.9%)	4010 (26.8%)	6676 (10.5%)	141,647 (44.5%)
Hypertension	74,082 (88.6%)	72,855 (99.4%)	81,502 (97.9%)	13,890 (92.9%)	60,897 (96.2%)	303,226 (95.2%)
Anemia	7609 (9.1%)	30,174 (41.2%)	33,804 (40.6%)	14,415 (96.4%)	10,224 (16.2%)	96,226 (30.2%)
Diabetes	281 (0.3%)	73,081 (99.7%)	0 (0%)	2597 (17.4%)	63,263 (100.0%)	139,222 (43.7%)
Obesity	2854 (3.4%)	25,323 (34.6%)	1688 (2.0%)	259 (1.7%)	36,812 (58.2%)	66,936 (21.0%)
Renal failure	3585 (4.3%)	31,489 (43.0%)	16,081 (19.3%)	6059 (40.5%)	9854 (15.6%)	67,068 (21.1%)
COPD	19,045 (22.8%)	37,795 (51.6%)	46,656 (56.0%)	4779 (31.9%)	18,113 (28.6%)	126,388 (39.7%)
Cancer	11,227 (13.4%)	18,097 (24.7%)	25,782 (31.0%)	4947 (33.1%)	7599 (12.0%)	67,652 (21.2%)
Liver disease	4506 (5.4%)	13,861 (18.9%)	8447 (10.1%)	3212 (21.5%)	8353 (13.2%)	38,379 (12.1%)
Peptic ulcer	2274 (2.7%)	5168 (7.1%)	5418 (6.5%)	1074 (7.2%)	2089 (3.3%)	16,023 (5.0%)
Dementia	4708 (5.6%)	5905 (8.1%)	10,913 (13.1%)	1897 (12.7%)	1567 (2.5%)	24,990 (7.8%)
Depression	7950 (9.5%)	15,019 (20.5%)	14,723 (17.7%)	3947 (26.4%)	9647 (15.2%)	51,286 (16.1%)
Alcohol misuse disorder	2335 (2.8%)	1862 (2.5%)	2845 (3.4%)	866 (5.8%)	1480 (2.3%)	9388 (2.9%)
**No. of comorbidities at baseline**						
2 or less	31,692 (37.9%)	0 (0%)	0 (0%)	374 (2.5%)	2773 (4.4%)	34,849 (10.9%)
3 to 4	46,386 (55.5%)	1413 (1.9%)	12,859 (15.4%)	6939 (46.4%)	27,130 (42.9%)	94,727 (29.8%)
5 to 6	5453 (6.5%)	23,548 (32.1%)	46,257 (55.5%)	6426 (43%)	27,668 (43.7%)	109,352 (34.3%)
7 to 8	46 (0.1%)	33,594 (45.8%)	20,804 (25%)	1151 (7.7%)	5540 (8.8%)	61,135 (19.2%)
Over 9	0 (0%)	14,729 (20.1%)	3363 (4%)	69 (0.5%)	160 (0.3%)	18,321 (5.8%)
**Inpatient diagnosis (vs. outpatient diagnosis)**	38,052 (45.5%)	40,079 (54.7%)	45,411 (54.5%)	8057 (53.9%)	31,845 (50.3%)	163,444 (51.3%)
**Business line**						
Medicare Advantage	50,158 (60.0%)	58,336 (79.6%)	63,766 (76.6%)	10,467 (70.0%)	37,752 (59.7%)	220,479 (69.2%)
Commercial	33,419 (40.0%)	14,948 (20.4%)	19,517 (23.4%)	4492 (30.0%)	25,529 (40.3%)	97,905 (30.8%)
**Race**						
White	60,104 (71.9%)	48,509 (66.2%)	61,596 (74.0%)	9517 (63.6%)	41,694 (65.9%)	221,420 (69.5%)
Black	10,277 (12.3%)	12,043 (16.4%)	9772 (11.7%)	2828 (18.9%)	11,322 (17.9%)	46,242 (14.5%)
Hispanic	5200 (6.2%)	6786 (9.3%)	4711 (5.7%)	1162 (7.8%)	4916 (7.8%)	22,775 (7.2%)
Asian	2064 (2.5%)	1332 (1.8%)	1619 (1.9%)	398 (2.7%)	947 (1.5%)	6360 (2.0%)
Missing	5932 (7.1%)	4614 (6.3%)	5585 (6.7%)	1054 (7.0%)	4402 (7.0%)	21,587 (6.8%)
**Education**						
Less than 12 grade	213 (0.3%)	333 (0.5%)	202 (0.2%)	50 (0.3%)	249 (0.4%)	1047 (0.3%)
High school diploma	26,027 (31.1%)	27,893 (38.1%)	27,614 (33.2%)	5239 (35.0%)	23,724 (37.5%)	110,497 (34.7%)
Less than bachelor degree	44,827 (53.6%)	37,696 (51.4%)	44,452 (53.4%)	7643 (51.1%)	33,086 (52.3%)	167,704 (52.7%)
Bachelor degree plus	11,994 (14.4%)	6811 (9.3%)	10,511 (12.6%)	1918 (12.8%)	5801 (9.2%)	37,035 (11.6%)
Missing	516 (0.6%)	551 (0.7%)	503 (0.6%)	109 (0.7%)	421 (0.7%)	2101 (0.7%)
**Income (in US dollars)**						
< $40,000	23,672 (28.3%)	25,282 (34.5%)	27,664 (33.2%)	4762 (31.8%)	20,137 (31.8%)	101,517 (31.9%)
$40,000–$74,000	21,529 (25.8%)	19,643 (26.8%)	21,849 (26.2%)	3600 (24.1%)	16,672 (26.3%)	83,293 (26.2%)
$75,000–$124,999	17,020 (20.4%)	12,704 (17.3%)	14,310 (17.2%)	2435 (16.3%)	12,621 (19.9%)	59,090 (18.6%)
$125,000–$199,999	6551 (7.8%)	3684 (5.0%)	4341 (5.2%)	891 (6.0%)	4102 (6.5%)	19,569 (6.1%)
$200,000+	3602 (4.3%)	1360 (1.9%)	1957 (2.3%)	423 (2.8%)	1642 (2.6%)	8984 (2.8%)
Missing	11,203 (13.4%)	10,611 (14.5%)	13,162 (15.8%)	2848 (19.0%)	8107 (12.8%)	45,931 (14.4%)
**Medication at baseline**						
Cardioselective beta-blockers	26,936 (32.2%)	32,949 (45.0%)	36,051 (43.3%)	4351 (29.1%)	22,095 (34.9%)	122,382 (38.4%)
Non-cardioselective beta-blockers	11,602 (13.9%)	13,169 (18.0%)	11,643 (14.0%)	1882 (12.6%)	9463 (15.0%)	47,759 (15.0%)
ACEi/ARBs	33,005 (39.5%)	41,120 (56.1%)	37,711 (45.3%)	5925 (39.6%)	31,073 (49.1%)	148,834 (46.7%)
MRA	3091 (3.7%)	3893 (5.3%)	2864 (3.4%)	733 (4.9%)	3233 (5.1%)	13,814 (4.3%)
Thiazide	9444 (11.3%)	12,071 (16.5%)	10,997 (13.2%)	1964 (13.1%)	9809 (15.5%)	44,285 (13.9%)
Loop diuretics	19,171 (22.9%)	28,629 (39.1%)	24,597 (29.5%)	4772 (31.9%)	21,361 (33.8%)	98,530 (30.9%)
Potassium-sparing diuretics	44 (0.1%)	90 (0.1%)	71 (0.1%)	23 (0.2%)	72 (0.1%)	300 (0.1%)
Double therapy (ACEi/ARBs + any beta-blocker)	19,842 (23.7%)	27,291 (37.2%)	24,207 (29.1%)	2990 (20.0%)	18,142 (28.7%)	92,472 (29.9%)
Triple therapy (ACEi/ARB + any beta blocker + MRA)	1530 (1.8%)	1905 (2.6%)	1273 (1.5%)	179 (1.2%)	1475 (2.3%)	6362 (2.0%)

*Abbreviations*: *IQR* inter-quartile range, *AF* atrial fibrillation, *CAD* coronary artery disease, *CVA* cerebrovascular disease, *PAD* peripheral artery disease, *COPD* chronic obstructive pulmonary disease, *No*. number, *US* United States, *ACEis* angiotensin-converting-enzyme inhibitors, *ARBs* angiotensin receptor blockers, *MRA* mineralocorticoid receptor antagonists

**Fig. 1:**
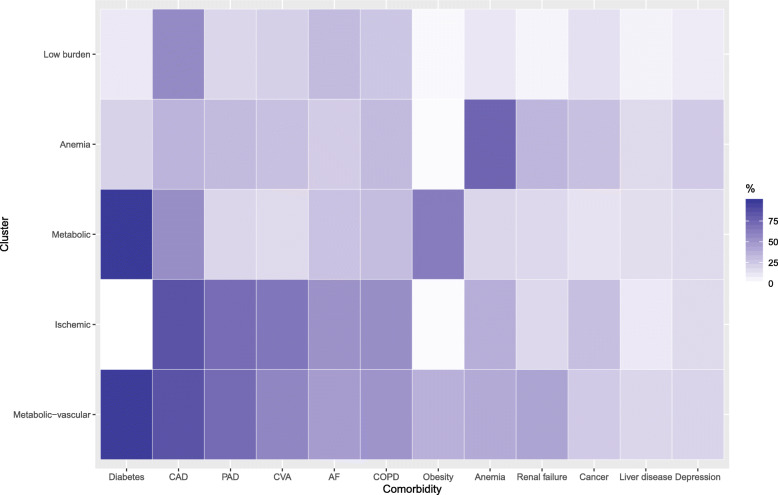
Five comorbidity clusters identified in patients with heart failure. Tile plot illustrating cluster-specific comorbidity percentages from the latent class analysis results. *CAD*, coronary artery disease; *PAD*, peripheral artery disease; *CVA*, cerebrovascular accident; *AF*, atrial fibrillation; *COPD*, chronic obstructive pulmonary disease

A five-group solution was the best fit to describe comorbidity patterns. The five clusters were each characterized by a different combination of comorbidities and socio-demographic factors and named according to the dominant features: low-burden, metabolic-vascular, ischemic, anemic, and metabolic (Fig. 1). Patients in the low-burden group had proportionately fewer comorbidities as compared to the other groups. Among these, CAD was most common (52.6% of patients). These patients were least likely to have received their HF diagnosis as an inpatient or to be on any HF medication. Almost all patients in the metabolic-vascular cluster had diabetes (99.7%) and 34.6% were obese. This group also had the highest prevalence of renal failure and patients on Medicare Advantage versus a commercial insurance plan. The metabolic-vascular group also had the highest percentage of HF prescriptions overall.

The ischemic cluster was the oldest group (median 78 years) and had no patients with diabetes, though a similarly high prevalence of CAD (88.2%), and PAD (76.9%) as the metabolic-vascular group and comparable proportion of patients with cardioselective beta-blocker prescriptions. The highest proportion of women (63.7%), cancer (33.1%), and depression (26.4%) was found in the anemic cluster. This group had an intermediate prescription rate for prognostic HF medications, as compared to the other clusters. Patients in the metabolic cluster were the youngest among clusters (median age 67 years), all were diabetic, and more than half were obese (58.2%). They also had the lowest prevalence of PAD (10.5%), CVA (13.1%), and cancer (12%), with intermediate prescription rates for HF medications (Table 1). Across all clusters, there was an increase in the number of patients who were prescribed HF medications from baseline to 1 year follow-up, except potassium-sparing diuretics. The highest increases were seen in MRA prescriptions, though levels were still low overall (between 8.2 and 12% of patients), followed by loop diuretics and beta-blockers (Additional file 1: Figure S3).

Ejection fraction (EF) group data were available in 13,560 patients (Additional file 1: Table S5) and smoking status in 35,721. Among those with EF data available, we observed the highest prevalence of HFpEF in the metabolic-vascular cluster. The prevalence of HFrEF was low in the anemic group but similar between other clusters (Additional file 1: Table S6).

### Clinical outcomes

**Fig. 2:**
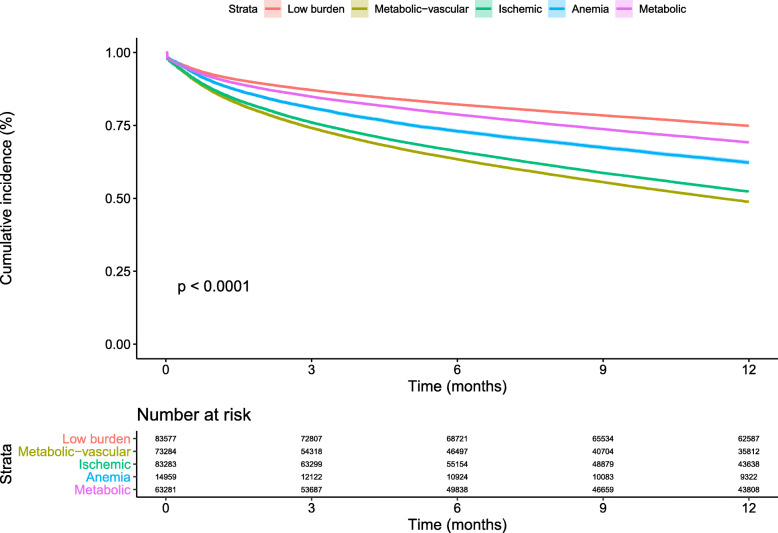
Kaplan-Meier curve showing difference for all-cause admission to hospital comorbidity clusters (within 1-year follow-up)

**Fig. 3:**
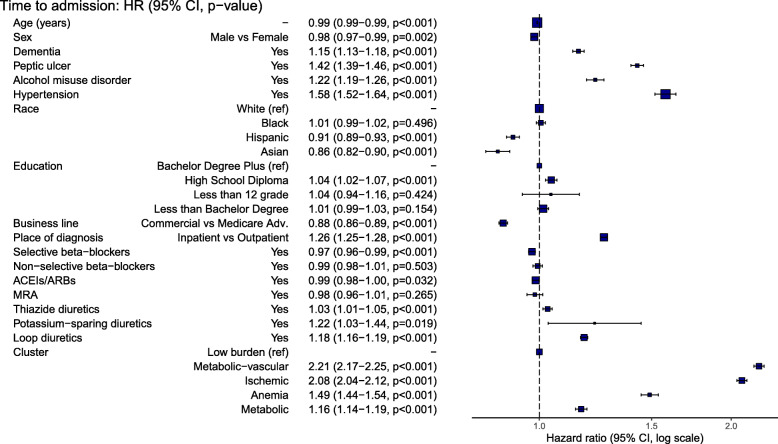
Cause-specific hazard ratios describing association between time to all-cause admission and comorbidity clusters, adjusted for baseline covariates, patients with missing data excluded (*N* = 295,972)

Overall, 38.7% of patients were admitted to hospital within the first year of follow-up after HF diagnosis; 8.8% were HF-specific admissions. A total of 25.1% of the low-burden group and 51.1% of the metabolic-vascular group experienced a hospitalization; the remaining groups had lower admission rates (Fig. 2, Additional file 1: Table S7). Differences in risk of admission persisted after adjusting for baseline covariates, with the lowest risk observed in the metabolic group and highest risk in the metabolic-vascular group, when compared to the low-burden group (Fig. 3). Differences remained significant when adjusting for EF and smoking status (Additional file 1: Table S8) and in sensitivity analysis accounting for uncertainty in class membership (Additional file 1: Table S9). The metabolic-vascular and ischemic clusters were associated with similarly high risk of HF-specific admission (increase of 85% and 81%, respectively) followed by the metabolic cluster (increase of 14%) (Additional file 1: Table S10). There was an increased risk of admission to hospital with increasing number of comorbidities (Additional file 1: Table S11).

Crude death rates were lowest in the metabolic and low-burden groups (3.76 per 1000 person-months [3.68–3.84] and 5.05 [4.97–5.12] respectively) and highest in the anemic (8.45 [8.21–8.70]) and ischemic groups (10.08 [9.96–10.18]). There was a statistically significant time-varying association between clusters and time-to-death for all except the anemic cluster (Additional file 1: Table S12, Figure S4). Interactions between cluster and EF are presented in Additional file 1: Table S13 and Table S14.

The metabolic-vascular group remained at the highest risk for death (adjusted HR 1.87, 95% CI 1.74–2.01) while the anemic and ischemic groups had intermediate risk (Table 2, Model 1). The metabolic group displayed the lowest risk of death among groups, and after adjustment for smoking status and EF, the estimate was not statistically significant (HR 0.96, 95% CI 0.84–1.10, *p* = 0.569).

**Table 2:** Association between any-cause mortality and comorbidity cluster

	Low-burden	Metabolic-vascular	Ischemic	Anemia	Metabolic	Overall
	*N* = 83,577	*N* = 73,284	*N* = 83,283	*N* = 14,959	*N* = 63,281	*N* = 318,030
**Deaths,** ***n*** **(%)**	18,497 (22.2%)	17,943 (24.5%)	32,709 (39.3%)	4615 (30.9%)	8774 (13.9%)	82,538 (26%)
**Person-months**	3,666,146	2,270,448	3,246,368	545,985	2,334,907	12,063,855
**Deaths per 1000 person-months (95% CI)**	5.05 (4.97–5.12)	7.9 (7.8–8.02)	10.08 (9.96–10.18)	8.45 (8.21–8.70)	3.76 (3.68–3.84)	6.84 (6.80–6.89)
**Model 1**^**a**^	*N* = 77,325	*N* = 68,414	*N* = 77,425	*N* = 13,850	*N* = 58,635	*N* = 295,649
**Deaths,** ***n*** **(%)**	17,291 (22.4%)	16,414 (24.7%)	30,510 (39.4%)	4325 (31.2%)	8251 (14.1%)	77,276 (26.1%)
**Adjusted HR**^**a**^ **(95% CI)**	1.00 (Ref)	1.87 (1.74–2.01)	1.24 (1.16–1.33)	1.46 (1.30–1.64)	1.18 (1.09–1.29)	
**Model 2**^**b**^	2707	3267	3265	247	2483	*N* = 12,091
**Deaths,** ***n*** **(%)**	510 (20.1%)	755 (24.6%)	1166 (38%)	98 (28.3%)	281 (12.1%)	3478 (28.8%)
**Adjusted HR**^**b**^ **(95% CI)**	1.00 (Ref)	1.60 (1.44, 1.79)	1.62 (1.47, 1.80)	1.60 (1.30, 1.96)	0.96 (0.84, 1.10)	

*Abbreviations*: *CI* confidence interval, *HR* hazard ratio, *ref*. reference

^a^Adjusted for age, sex, race, education, medical insurance status, whether diagnosis was gained inpatient or in outpatient and HF medications; time-varying coefficient model; excludes patients with missing data on race (21,557) and education (2097)

^b^Adjusted for age, sex, race, education, medical insurance status, whether diagnosis was gained inpatient or in outpatient, HF medications, ejection fraction, smoking status; proportional hazards met; excludes patients with missing data on race (21,557), education (2097), ejection fraction (304,477), and smoking status (282,333)

### Healthcare resource use

In adjusted analyses, all comorbidity clusters exhibited significantly increased rate ratios of healthcare utilization, when compared to the low-burden group. The metabolic-vascular and ischemic clusters had the highest rates of hospitalizations and associated cumulative length of stay, long-term care stays, and office visits, while the anemic group experienced the highest incidence rate of outpatient visits (Table 3). Cost differences mirrored healthcare utilization, with the metabolic-vascular cluster exhibiting the highest healthcare costs, followed by the ischemic, anemic, metabolic, and low-burden groups (Additional file 1: Table S15).

**Table 3:** Association between healthcare utilization and comorbidity cluster within 1 year of HF diagnosis

	Rate ratio (95% CI)		
	Unadjusted RR (95% CI)	Model 1^**a**^	Model 2^**b**^
	*N* = 314,936	*N* = 292,768	*N* = 11,955
Outcome and comorbidity cluster			
**Outpatient visits**			
Low-burden	Ref.	Ref.	Ref.
Metabolic-vascular	2.33 (2.30, 2.36)	2.01 (1.98, 2.04)	1.96 (1.84, 2.08)
Ischemic	1.91 (1.89, 1.93)	1.73 (1.71, 1.75)	1.70 (1.60, 1.81)
Anemia	2.32 (2.26, 2.37)	2.11 (2.06, 2.16)	1.89 (1.67, 2.14)
Metabolic	1.24 (1.22, 1.25)	1.17 (1.15, 1.20)	1.13 (1.06, 1.21)
**Office visits**			
Low-burden	Ref.	Ref.	Ref.
Metabolic-vascular	1.29 (1.28, 1.31)	1.32 (1.31, 1.33)	1.23 (1.17, 1.28)
Ischemic	1.30 (1.29, 1.31)	1.35 (1.34, 1.37)	1.28 (1.23, 1.34)
Anemia	1.15 (1.13, 1.17)	1.16 (1.15, 1.18)	1.26 (1.16, 1.38)
Metabolic	1.08 (1.07, 1.09)	1.05 (1.04, 1.06)	1.05 (1.00, 1.10)
**Long-term care stays**			
Low-burden	Ref.	Ref.	Ref.
Metabolic-vascular	2.87 (2.78, 2.96)	2.54 (2.46, 2.62)	2.75 (2.32, 3.26)
Ischemic	3.06 (2.95, 3.14)	2.26 (2.19, 2.33)	2.38 (2.01, 2.82)
Anemia	2.22 (2.10, 2.33)	1.77 (1.67, 1.86)	2.41 (1.81, 3.21)
Metabolic	1.02 (0.98, 1.06)	1.21 (1.17, 1.26)	1.12 (0.90, 1.38)
**Hospitalizations**			
Low-burden	Ref.	Ref.	Ref.
Metabolic-vascular	2.86 (2.78, 2.96)	2.11 (2.08, 2.15)	2.02 (1.86, 2.19)
Anemia	2.22 (2.78, 2.96)	1.64 (1.59, 1.68)	1.85 (1.59, 2.16)
Ischemia	3.04 (2.95, 3.14)	2.11 (2.07, 2.15)	1.99 (1.83, 2.17)
Metabolic	1.01 (0.97, 1.05)	1.07 (1.04, 1.09)	1.09 (0.99, 1.19)
**Length of stay for hospitalizations**			
Low-burden	Ref.	Ref.	Ref.
Metabolic-vascular	2.70 (2.97, 3.08)	2.58 (2.52, 2.65)	2.60 (2.28, 2.95)
Ischemic	2.43 (2.37, 2.49)	2.48 (2.41, 2.54)	2.44 (2.14, 2.77)
Anemia	2.39 (2.29, 2.50)	2.08 (2.01, 2.16)	2.29 (1.85, 2.80)
Metabolic	1.25 (1.21, 1.27)	1.09 (1.06, 1.13)	1.17 (1.00, 1.35)

*Abbreviations*: *CI* confidence interval, *RR* rate ratio, *ref*. reference

^a^Adjusted for age, sex, race, education, medical insurance status, whether diagnosis was gained inpatient or in outpatient and HF medications; patients with missing data were excluded

^b^Adjusted for age, sex, race, education, medical insurance status, whether diagnosis was gained inpatient or in outpatient and HF medications, ejection fraction, smoking status; patients with missing data were excluded

## Discussion

To our knowledge, this is the largest study of model-based clustering in HF published to date, using widely available clinical variables and a population sample which is representative of people living in the US. In doing so, we identified five distinct comorbidity clusters of patients with HF, namely the low-burden, metabolic-vascular, anemic, ischemic, and metabolic groups. Importantly, these comorbidity clusters had differential risks of hospital admission and death, indicating that comorbidity patterns reflect variable HF clinical trajectories and prognosis.

Previous studies have identified subgroups in HF: Tromp et al. [11] included registry patients from across Asia and identified five clusters, which had differential quality of life and rates of a combined outcome of death or HF hospitalization over 1 year follow-up. They similarly identified ischemic and metabolic subgroups, but with markedly different characteristics to the current cohort. Notably, the Asian metabolic group had lower rates of both diabetes (63.5% vs. 100%) and obesity (45.1% vs. 58%) and was on average 10 years younger than the US group. The Asian ischemic cluster had comparable prevalence of CAD; however, the US group had a higher frequency of non-CV comorbidities such as cancer and liver disease. The remaining three clusters identified by Tromp et al. [11], elderly/AF, young, and lean diabetic, did not have direct equivalence in the US, suggesting clustering of comorbidities may be specific to geographical region.

Another study, from the US, found four subgroups in a hospitalized HF population: a common disease group characterized by high prevalence of hypertension, a lifestyle group with high diabetes and obesity, a renal group, and a neurovascular group with increased levels of cerebrovascular disease [10]. The latter group was at most increased risk of inpatient mortality and had the highest medical cost. However, this cohort may reflect a more severe population as only hospitalized patients were included and was further limited by solely examining inpatient outcomes.

In our population-wide study, we found two new US-specific clusters: the anemic and metabolic-vascular groups. It is the first time a principally anemic group has been identified using model-based clustering techniques in HF. The second most frequently diagnosed comorbidity in this group was renal failure, with a prevalence second only to the metabolic-vascular group. Thus, it is not surprising that these two comorbidities clustered together, as the cardio-renal anemia syndrome is well-established in HF and has been linked to increased hospitalization and worse clinical prognosis as compared to patients without these comorbidities [25–27]. Compared to the low-burden cluster, the anemic group was at increased risk of both admission and mortality (49% and 46% increased risk, respectively). Surprisingly, the risk of death in this group was numerically higher than for patients in the ischemic group, suggesting this triad of comorbidities (HF, anemia, renal failure) incurs a higher clinical burden than that of patients fitting an older profile with more CV disease (such as the ischemic group).

Patients in the metabolic-vascular phenotype had the worst prognosis, denoted by the highest risk of admission and death compared to the low-burden group. The association with admission was significant after adjusting for HF medications, suggesting that therapies aimed at modifying mortality and morbidity risk and congestion relief do not necessarily decrease admission risk in this patient group. Although we did not assess compliance with medical or management of comorbidities, the particular combination of high-risk CV (PAD, CAD) and non-CV comorbidities (renal failure, diabetes) may increase the risk of admission independent of these factors.

The metabolic group had the lowest risk of admission or death, despite all patients being diagnosed with diabetes and over half with obesity. This group was, on average, the youngest among clusters, which may explain the comparatively favorable prognosis. Other studies [28, 29] have reported on the “obesity paradox” in HF where higher BMI appears to act as a protective factor against mortality or admission, though this has been described as either wrongly diagnosing HF in obese individuals, or lead time bias (earlier symptom onset attributable to added metabolic demands of obesity/diabetes), which may be plausible in a younger HF subgroup.

Nearly two thirds of our overall cohort had five or more comorbidities, similar to previous reports [30]. The total number of comorbidities varied across clusters and was highest in those with the poorest prognosis (i.e., metabolic-vascular, ischemic subgroups), confirming that increases in comorbidity burden worsen prognosis. Furthermore, there was a stepwise increase in risk of admission to hospital with each incremental rise in number of comorbidities, and those with over nine comorbidities were at tripled risk of being admitted to hospital, compared to those with two or less additional illnesses (Additional file 1: Table S11). However, individual comorbidity counts insufficiently describe the differences in clinical burden incurred by comorbid diseases (for example, anemia may be associated with a lower level of disability as compared to CAD, but the two diseases contribute equally when using a counting approach). Individual comorbidity counts may also fail to convey the severity of diseases or interactions between comorbidities that may give rise to distinct clinical trajectories. By contrast, identification of specific patterns or clusters of comorbidities, as performed in our study, may capture some of these interactions and provide more granular information that could identify priorities for clinical HF care.

Furthermore, among patients with EF data available, although we observed some preferential distribution of HFpEF to the metabolic-vascular or ischemic groups, and a greater predominance of HFrEF in the low-burden group, none of the clusters mapped perfectly to either EF group, highlighting the complexity and interrelatedness of comorbidity in HF (Additional file 1: Table S6) [31]. Importantly, differences in admission and survival persisted after adjusting for EF, which also did not act as an effect modifier (Additional file 1: Table S8, Table S13, Table S14), corroborating previous research showing that most comorbidities have a similar impact on both EF-defined HF groups [32]. Although EF has been the primary framework used to classify patients with HF, and the basis for recruitment into therapeutic trials, there are still no proven disease-modifying treatments for up to half of all patients with HF—i.e., those with preserved EF. Our findings suggest a potential for clinical trials to enroll patients and test therapies based on prognostic comorbidity patterns, not just limiting them to EF.

Healthcare resource utilization has not previously been reported in clustering studies of HF. Our data demonstrate a significant association of comorbidity patterns with healthcare utilization in HF. We found that patients with higher prevalence of CV comorbidities (metabolic-vascular, ischemic) were more often admitted to hospital, in contrast to the metabolic and anemic patients, who had comparatively more outpatient visits during follow-up. The lowest utilization rate was observed in the metabolic group. This may partly be explained by the younger age of patients in this group, and/or a low requirement for healthcare use for metabolic conditions in the absence of vascular complications (i.e., no CAD, PAD, and CVA). These data demonstrate a significant association of comorbidity patterns with healthcare utilization in HF and may reflect the different intensity of care and surveillance needed for the management of specific comorbidities or variable severity of associated HF across the clusters.

The anemic cluster experienced the highest adjusted rate of outpatient visits and high mortality. The main distinguishing features of this cluster (namely anemia-depression-cancer) have been independently linked to increased use of outpatient services, explained partly by care-seeking behaviors, poor medication adherence in depression [33], or undertreatment of HF due to deteriorating in health status in malignancy [34]. Indeed, the anemic cluster had among the lowest proportions of medication prescriptions across clusters, suggesting less than optimal management of HF.

Cost of care was primarily driven by inpatient and emergency room visits and was highest in the metabolic-vascular profile, intermediate in the anemic and ischemic groups, and lowest in the metabolic and low-burden groups, respectively. The identification of this “hierarchy” of cost, associated with common comorbidity patterns, calls for a targeted approach of resource allocation: thus, patients fitting profiles characterized by high inpatient use should be the focus of community interventions targeting lifestyle changes such as providing nutritional advice, encouraging exercise regimens, and compliance with HF medication that may help to prevent admissions to hospital.

Overall, it is challenging to manage patients with HF with co-occurring disease. Our results emphasize that the specific knowledge of how comorbidities cluster together and their association with clinical prognosis may assist clinicians who manage these complex patients to further refine and target their treatment. Arguably, patients within each cluster are more similar, on a group level, compared to those in other clusters—whether these subgroups may benefit from similar preventative and therapeutic plans needs to be evaluated in future prospective studies. Future characterizations of HF may benefit from integrating data on comorbidities ideally derived from large, real-world populations in relevant and local geographical settings, in order to derive a more nuanced taxonomy, enabling multidimensional and personalized HF care and resource allocation. Furthermore, our clustering analysis may serve as a hypothesis-generating paradigm in identifying comorbidity patterns, which may be improved upon in further studies. It would be interesting to assess whether membership to comorbidity cluster changes over time in patients with HF and to map their trajectories, similar to Vetrano and colleagues, who evaluated elderly individuals’ transitions among multimorbidity clusters over time [35]. A controlled setting such as a registry where data collection is standardized and collected at specific time points by trained healthcare staff may be more suitable for such an investigation.

## Strengths and limitations

We included a large number of patients with incident HF from the US, reflective of those who are commercially insured or on Medicare Advantage, unlike previous studies with small sample sizes and restricted inclusion criteria. The prevalence of specific risk factors for HF, such as hypertension and CAD, was marginally higher compared to other studies of HF [36, 37]. However, we included patients from across the US: of all ages, ethnic groups, and both sexes, with a similar distribution to other large national studies [38, 39].

There are a number of limitations: diagnoses were based on ICD codes only, though these have been validated [40, 41]. The use of administrative data means diagnoses can be subject to misclassification and measurement error. However, by linking outpatient and hospital claims, we were able to identify the date of incident HF and assess comorbidities which were diagnosed prior to this, limiting the inclusion of cases where precursors of HF may have been incorrectly labeled as HF. Furthermore, changes in diagnostic procedures over time, specific to HF, such as improvements in echocardiography, might have increased likelihood of detecting milder forms of the disease in more recent times, which would be difficult to assess.

We did not have data on severity of HF or control of comorbidities; however, in outcome analyses, we adjusted for use of diuretics, which may be considered a surrogate for the presence of congestion. Due to changes in recording of mortality in the OLDW databases in recent years, we were limited in the possibility of undertaking a competing risk analysis for the main outcome and thus investigate whether risk of admission to hospital may be overestimated in our study. Despite the potentially incomplete mortality data, we have performed an analysis of the risk of admission to hospital, accounting for the competing risk of death within the first year of HF diagnosis, which shows a similar result to the main analysis (Additional file 1: Table S16 [42, 43]).

Finally, the aim of our analysis was not to create a novel prediction model for outcomes in HF, which already exist and have been validated. The approach used to derive the comorbidity clusters was unbiased, i.e., data-driven with no a priori theory applied on how we expected the comorbidities to cluster. This was designed to identify novel, potentially “hidden” patterns that may guide clinical management and resource allocation in a real-world setting, but concurrently identified prognostic differences. HF patients typically present with a constellation of characteristics which overlap—this is reflected in our analysis where several comorbidities were observed across the five identified clusters, albeit in different proportions.

## Conclusion

In this large cohort of patients with HF from the US, we have demonstrated that electronic healthcare record data may be used to generate a more granular classification of HF, based on comorbidities and their combinations. We identified five comorbidity clusters that exhibited differences in the risks of hospital admission, mortality, and healthcare resource utilization. These findings suggest an opportunity for future RCTs to incorporate comorbidity patterns in their enrollment criteria and a need for tailored comorbidity management and prevention plans to accompany existing evidence-based medical therapy for patients with HF, in particular targeting the clusters with the poorest prognosis.

## Supplementary Information

**Additional file 1: Supplemental Methods.** Population. Latent Class Analysis. Descriptive Statistics. Negative Binomial Regressions. HF-specific admission and mortality analyses. Sensitivity analysis – pseudo-class draws. **Figure S1.** Fit indices for the 2 to 9 class solution models derived using latent class analysis. **Figure S2.** Partial probabilities of class membership for all variables used in deriving the clusters. **Figure S3.** Prescription patterns for heart failure recommended medications as well as diuretics, from baseline to one-year follow-up, across comorbidity clusters. **Figure S4.** Hazard ratios (95%CI) for mortality per time group, according to comorbidity cluster. **Table S1.** List of ICD9 and ICD10 codes used to identify heart failure patients. **Table S2.** Medication classes captured from pharmacy claims. **Table S3**. Fit statistics for 2-9 latent class models. **Table S4.** Median (IQR) probability of group membership for the 5-class solution. **Table S5.** Baseline characteristics according to ejection fraction group, in patients with data available. **Table S6.** Distribution of ejection fraction group and smoking status across comorbidity clusters in patients with data available. **Table S7.** Frequency of admission to hospital across comorbidity clusters. **Table S8.** Association between admission to hospital and comorbidity clusters, adjusted for ejection fraction and smoking status. **Table S9.** Sensitivity analysis results - adjusted association between admission to hospital and comorbidity cluster. Results from 20 models using imputed class assignments. **Table S10.** Association between heart failure-specific admission to hospital and comorbidity clusters.**Table S11.** Association between admission to hospital and number of comorbidities**. Table S12.** Association between mortality and comorbidity cluster with interaction between cluster and time. **Table S13.** Association between mortality and comorbidity cluster with interaction between ejection fraction and cluster. **Table S14.** Association between mortality and comorbidity cluster with interaction between ejection fraction and cluster. **Table S15.** Costs associated with healthcare resource use, per comorbidity cluster. **Table S16.** Competing risk analysis.

## Data Availability

The data that support the findings of this study are available from OptumLabs, but restrictions apply to the availability of these data, which were used under license for the current study, and so are not publicly available. Data are however available from the authors upon reasonable request and with permission of OptumLabs. Access to these data is only available through entering into an exclusive institutional partnership agreement with OptumLabs, under which this study was conducted.

## Abbreviations

ACEi: Angiotensin-converting-enzyme inhibitor
AF: Atrial fibrillation
ARB: Angiotensin receptor blocker
CAD: Coronary artery disease
COPD: Chronic obstructive pulmonary disease
CVA: Cerebrovascular disease
EF: Ejection fraction
HF: Heart failure
HFrEF: Heart failure with reduced ejection fraction
HFmEF: Heart failure with mid-range ejection fraction
HFpEF: Heart failure with preserved ejection fraction
LCA: Latent class analysis
MRA: Mineralocorticoid receptor antagonists
PAD: Peripheral artery disease

## References

[1] Ponikowski P, Voors AA, Anker SD, Bueno H, Cleland JG, Coats AJ (2016). 2016 ESC Guidelines for the diagnosis and treatment of acute and chronic heart failure: the Task Force for the diagnosis and treatment of acute and chronic heart failure of the European Society of Cardiology (ESC) developed with the special contribution of the Heart Failure Association (HFA) of the ESC. Eur Heart J.

[2] Yancy CW, Jessup M, Bozkurt B, Butler J, Casey DE, Colvin MM (2017). 2017 ACC/AHA/HFSA focused update of the 2013 ACCF/AHA guideline for the management of heart failure: a report of the American College of Cardiology/American Heart Association Task Force on Clinical Practice Guidelines and the Heart Failure Society of America. J Am Coll Cardiol.

[3] Bui AL, Horwich TB, Fonarow GC (2011). Epidemiology and risk profile of heart failure. Nat Rev Cardiol.

[4] Chamberlain AM, Boyd CM, Manemann SM, Dunlay SM, Gerber Y, Killian JM (2020). Risk factors for heart failure in the community: differences by age and ejection fraction. Am J Med.

[5] He J, Ogden LG, Bazzano LA, Vupputuri S, Loria C, Whelton PK (2001). Risk factors for congestive heart failure in US men and women: NHANES I epidemiologic follow-up study. Arch Intern Med.

[6] Komanduri S, Jadhao Y, Guduru SS, Cheriyath P, Wert Y (2017). Prevalence and risk factors of heart failure in the USA: NHANES 2013 - 2014 epidemiological follow-up study. J Community Hosp Intern Med Perspect.

[7] Lawson CA, Solis-Trapala I, Dahlstrom U, Mamas M, Jaarsma T, Kadam UT (2018). Comorbidity health pathways in heart failure patients: a sequences-of-regressions analysis using cross-sectional data from 10,575 patients in the Swedish Heart Failure Registry. PLoS Med.

[8] Lawson CA, Zaccardi F, Squire I, Okhai H, Davies M, Huang W (2020). Risk factors for heart failure: 20-year population-based trends by sex, socioeconomic status, and ethnicity. Circ Heart Fail..

[9] Horiuchi Y, Tanimoto S, Latif A, Urayama KY, Aoki J, Yahagi K (2018). Identifying novel phenotypes of acute heart failure using cluster analysis of clinical variables. Int J Cardiol.

[10] Lee CS, Chien CV, Bidwell JT, Gelow JM, Denfeld QE, Creber RM (2014). Comorbidity profiles and inpatient outcomes during hospitalization for heart failure: an analysis of the US Nationwide inpatient sample. BMC Cardiovasc Disord.

[11] Tromp J, Tay WT, Ouwerkerk W, Teng TK, Yap J, MacDonald MR (2018). Multimorbidity in patients with heart failure from 11 Asian regions: a prospective cohort study using the ASIAN-HF registry. PLoS Med.

[12] Hedman AK, Hage C, Sharma A, Brosnan MJ, Buckbinder L, Gan LM (2020). Identification of novel pheno-groups in heart failure with preserved ejection fraction using machine learning. Heart..

[13] Shah SJ, Katz DH, Selvaraj S, Burke MA, Yancy CW, Gheorghiade M (2015). Phenomapping for novel classification of heart failure with preserved ejection fraction. Circulation..

[14] Ferreira JP, Duarte K, McMurray JJV, Pitt B, van Veldhuisen DJ, Vincent J (2018). Data-driven approach to identify subgroups of heart failure with reduced ejection fraction patients with different prognoses and aldosterone antagonist response patterns. Circ Heart Fail..

[15] Kao DP, Lewsey JD, Anand IS, Massie BM, Zile MR, Carson PE (2015). Characterization of subgroups of heart failure patients with preserved ejection fraction with possible implications for prognosis and treatment response. Eur J Heart Fail.

[16] Kao DP, Wagner BD, Robertson AD, Bristow MR, Lowes BD (2012). A personalized BEST: characterization of latent clinical classes of nonischemic heart failure that predict outcomes and response to bucindolol. PLoS One.

[17] Segar MW, Patel KV, Ayers C, Basit M, Tang WHW, Willett D (2020). Phenomapping of patients with heart failure with preserved ejection fraction using machine learning-based unsupervised cluster analysis. Eur J Heart Fail.

[18] OptumLabs (2019). OptumLabs and OptumLabs Data Warehouse (OLDW) Descriptions and Citation.

[19] Linzer DA, Lewis JB (2011). poLCA: an R package for polytomous variable latent class analysis. J Stat Softw.

[20] Wang C-P, Hendricks Brown C, Bandeen-Roche K (2005). Residual diagnostics for growth mixture models: examining the impact of a preventive intervention on multiple trajectories of aggressive behavior. J Am Stat Assoc.

[21] Rubin DB (2004). Multiple imputation for nonresponse in surveys: John Wiley & Sons.

[22] Schoenfeld D (1982). Partial residuals for the proportional hazards regression model. Biometrika..

[23] Therneau T, Crowson C, Atkinson E. Using time dependent covariates and time dependent coefficients in the cox model. Survival Vignettes. 2017.

[24] Gulea C. Analysis code for the results presented in: Claudia Gulea, Rosita Zakeri, Jennifer K. Quint: “Model-based comorbidity clusters in patients with heart failure: association with clinical outcomes and healthcare utilization”. ZENODO DOI. 10.5281/zenodo.4278086. 10.1186/s12916-020-01881-7 PMC7812726 33455580

[25] Anand IS, Gupta P (2018). Anemia and iron deficiency in heart failure: current concepts and emerging therapies. Circulation..

[26] Grote Beverborg N, van Veldhuisen DJ, van der Meer P (2018). Anemia in heart failure: still relevant?. JACC Heart Fail.

[27] Silverberg DS, Wexler D, Blum M, Iaina A, Sheps D, Keren G (2005). Erythropoietin in heart failure. Semin Nephrol.

[28] Güder G, Frantz S, Bauersachs J, Allolio B, Wanner C, Koller MT, et al. Reverse epidemiology in systolic and nonsystolic heart failure: cumulative prognostic benefit of classical cardiovascular risk factors. Circ Heart Fail. 2009;2(6):563–71. 10.1161/CIRCHEARTFAILURE.108.825059 19919981

[29] Lavie CJ, Mehra MR, Milani RV. Obesity and heart failure prognosis: paradox or reverse epidemiology? Eur Heart J. 2004;26(1):5–7. 10.1093/eurheartj/ehi055 15615792

[30] Wong CY, Chaudhry SI, Desai MM, Krumholz HM (2011). Trends in comorbidity, disability, and polypharmacy in heart failure. Am J Med.

[31] Triposkiadis F, Giamouzis G, Parissis J, Starling RC, Boudoulas H, Skoularigis J (2016). Reframing the association and significance of co-morbidities in heart failure. Eur J Heart Fail.

[32] Ather S, Chan W, Bozkurt B, Aguilar D, Ramasubbu K, Zachariah AA (2012). Impact of noncardiac comorbidities on morbidity and mortality in a predominantly male population with heart failure and preserved versus reduced ejection fraction. J Am Coll Cardiol.

[33] Moraska AR, Chamberlain AM, Shah ND, Vickers KS, Rummans TA, Dunlay SM (2013). Depression, healthcare utilization, and death in heart failure: a community study. Circ Heart Fail.

[34] Meijers WC, de Boer RA (2019). Common risk factors for heart failure and cancer. Cardiovasc Res.

[35] Vetrano DL, Roso-Llorach A, Fernandez S, Guisado-Clavero M, Violan C, Onder G (2020). Twelve-year clinical trajectories of multimorbidity in a population of older adults. Nat Commun.

[36] Chamberlain AM, St Sauver JL, Gerber Y, Manemann SM, Boyd CM, Dunlay SM (2015). Multimorbidity in heart failure: a community perspective. Am J Med.

[37] Roger VL (2013). Epidemiology of heart failure. Circ Res.

[38] Forman DE, Cannon CP, Hernandez AF, Liang L, Yancy C, Fonarow GC (2009). Influence of age on the management of heart failure: findings from Get With the Guidelines-Heart Failure (GWTG-HF). Am Heart J.

[39] Sangaralingham LR, Shah ND, Yao X, Roger VL, Dunlay SM (2016). Incidence and early outcomes of heart failure in commercially insured and Medicare Advantage patients, 2006 to 2014. Circ Cardiovasc Qual Outcomes.

[40] Li Q, Glynn RJ, Dreyer NA, Liu J, Mogun H, Setoguchi S (2011). Validity of claims-based definitions of left ventricular systolic dysfunction in Medicare patients. Pharmacoepidemiol Drug Saf.

[41] Quach S, Blais C, Quan H (2010). Administrative data have high variation in validity for recording heart failure. Can J Cardiol.

[42] Fine JP, Gray RJ (1999). A proportional hazards model for the subdistribution of a competing risk. J Am Stat Assoc.

[43] Gray B (2020). cmprsk: subdistribution analysis of competing risks. R package version 2.2-10.

