# Supplementary material for: Model-based comorbidity clusters in patients with heart failure: association with clinical outcomes and healthcare utilization
Source: BMC Med. 2021 Jan 18;19:9. doi: 10.1186/s12916-020-01881-7 (PMC7812726; doi:10.1186/s12916-020-01881-7)
Supplement: Supplementary file 1 — Additional file 1: Supplemental Methods. Population. Latent Class Analysis. Descriptive Statistics. Negative Binomial Regressions. HF-specific admission and mortality analyses. Sensitivity analysis – pseudo-class draws. Figure S1. Fit indices for the 2 to 9 class solution models derived using latent class analysis. Figure S2. Partial probabilities of class membership for all variables used in deriving the clusters. Figure S3. Prescription patterns for heart failure recommended medications as well as diuretics, from baseline to one-year follow-up, across comorbidity clusters. Figure S4. Hazard ratios (95%CI) for mortality per time group, according to comorbidity cluster. Table S1. List of ICD9 and ICD10 codes used to identify heart failure patients. Table S2. Medication classes captured from pharmacy claims. Table S3. Fit statistics for 2-9 latent class models. Table S4. Median (IQR) probability of group membership for the 5-class solution. Table S5. Baseline characteristics according to ejection fraction group, in patients with data available. Table S6. Distribution of ejection fraction group and smoking status across comorbidity clusters in patients with data available. Table S7. Frequency of admission to hospital across comorbidity clusters. Table S8. Association between admission to hospital and comorbidity clusters, adjusted for ejection fraction and smoking status. Table S9. Sensitivity analysis results - adjusted association between admission to hospital and comorbidity cluster. Results from 20 models using imputed class assignments. Table S10. Association between heart failure-specific admission to hospital and comorbidity clusters.Table S11. Association between admission to hospital and number of comorbidities. Table S12. Association between mortality and comorbidity cluster with interaction between cluster and time. Table S13. Association between mortality and comorbidity cluster with interaction between ejection fraction and cluster. Table S14. Association [file 12916_2020_1881_MOESM1_ESM.docx]

**Supplement**

**Supplemental Methods**

**Population**……………………………………………………………………………………….3

**Latent class analysis**………………………………………………………………………….3

**Descriptive statistics**…………………………………………………………………………..4

**Negative binomial regressions**………………………………………………………………4

**HF-specific admission and mortality analyses**……………………………………………4

**Sensitivity analysis – pseudo-class draws** ……………………………………………….4

**Figure S1**. Fit indices for the 2 to 9 class solution models derived using latent class analysis….6

**Figure S2.** Partial probabilities of class membership for all variables used in deriving the clusters, per class………………………………………………………………………………………...7

**Figure S3**. Prescription patterns for heart failure recommended medications as well as diuretics, from baseline to one-year follow-up, across comorbidity clusters (potassium-sparing not shown due to negligible proportions)……………………………………………………………………………8

**Figure S4.** Hazard ratios (95%CI) for mortality per time group, according to comorbidity cluster………………………………………………………………………………………………………9

**Table S1.** List of ICD9 and ICD10 codes used to identify heart failure patients…………………10

**Table S2.** Medication classes captured from pharmacy claims……………………………………11

**Table S3**. Fit statistics for 2-9 latent class models………………………………………………….12

**Table S4.** Median (IQR) probability of group membership for the 5-class solution……………...13

**Table S5.** Baseline characteristics according to ejection fraction group, in patients with data available (N=13560)…………………………………………………………………………………….14

**Table S6.** Distribution of ejection fraction group and smoking status across comorbidity clusters in patients with data available………………………………………………………………………….18

**Table S7.** Frequency of admission to hospital across comorbidity clusters, within one-year follow-up……………………………………………………………………………………………19

**Table S8.** Association between admission to hospital and comorbidity clusters, adjusted for ejection fraction and smoking status, within one-year follow-up (N=11294)…………………………………………….………………………………………………….20

**Table S9.** Sensitivity analysis results - adjusted association between admission to hospital and comorbidity cluster, within one-year follow-up. Results from 20 models using imputed class assignments (estimates combined using Rubin’s rule) and results from the main analysis………………………………………………………………………………………………..…23

**Table S10.** Association between heart failure-specific admission to hospital and comorbidity clusters, with time-varying coefficient due to non-proportional hazards, within one-year follow-up…………………………………………………………………………….…………………………...24

**Table S11.** Association between admission to hospital and number of comorbidities, within one-year follow-up……………………………………………………………………………………………25

**Table S12.** Association between mortality and comorbidity cluster with interaction between cluster and time (median 30 months follow-up)……………………………………………………...26

**Table S13.** Association between mortality and comorbidity cluster with interaction between ejection fraction and cluster (proportional hazards met)……………………………………………28

**Table S14.** Association between mortality and comorbidity cluster with interaction between ejection fraction and cluster, including coefficients and standard errors for interaction terms….30

**Table S15.** Costs associated with healthcare resource use, per comorbidity cluster, within one-year follow-up (currency United States dollars $) …………………………………………………………………………………………………………….31

**Table S16.** Competing risk analysis (death as competing risk to hospitalization) ...………………………………………………………………………………………………………….32

**Supplemental Methods**

**Population**

Given that hypertension and coronary artery disease (CAD) are two of the most common causes of heart failure (HF), we expected that some patients identified with incident HF (due to these conditions) would have already been prescribed some of the medications of interest (e.g. angiotensin-converting-enzyme [ACE] inhibitors and beta-blockers). We assumed some of these patients would not receive new prescriptions immediately after a diagnosis of HF, but de facto, still use the medications. In order to ensure we identified all relevant medications at HF diagnosis, pharmacological treatments were also captured in the baseline period.

**Latent class analysis**

We used R package “poLCA” to perform latent class analysis (LCA) in order to identify clusters of comorbidities in patients with HF. LCA is a model-based clustering technique that classifies individuals into subgroups based on multiple characteristics in a cohort (in this case comorbidities). We used a comprehensive list of comorbidities taking into account known associations with HF. Comorbidity variables used to derive the clusters were: atrial fibrillation (AF), anemia, CAD, cancer, chronic obstructive pulmonary disease (COPD), cerebrovascular accident (CVA), diabetes mellitus, depression, liver disease, obesity, peripheral artery disease (PAD) and renal failure. Hypertension, alcohol misuse disorder, dementia and peptic ulcer were not used in the main LCA model as they were unlikely to discriminate subpopulations of patients (due to homogeneity in the former (95.2%) and small prevalence in the latter three characteristics (2.9%, 7.8%, respectively 5%). Age, sex and sociodemographic variables were not used in the LCA but we adjusted for these in subsequent analyses.

Maximum-likelihood estimation was used to identify clusters for a range of 2 to 9 groups. Cluster membership was based on parametric estimates of grouping individuals (compared different models and best solution based on statistics and clinical interpretability). This approach is more robust over other distance-based clustering techniques as it permits a mathematical evaluation of how well a model represents the data.

The metrics used to determine the best cluster solution were based on the following criteria:

- Information criteria such as the Bayesian Information Criterion (BIC), sample adjusted BIC and Akaike Information Criterion (AIC) and log-likelihood where lower values indicate superior fit. While the fit indices continue to improve beyond the six-class solution, the incremental improvement in fit was not large enough to account for the increase in complexity of interpretation (Figure S1).
- No small classes: the rule of thumb is that there is no cluster size below 5% of the overall study population (generally, this may represent a “left-over” class – of patients that cannot be assigned with confidence to any other class). In our case, choosing 6 cluster would result in one cluster making up only 3.9% of the total cohort.
- Clinical interpretability – the five class solution distinguished clinically relevant clusters, some of which have equivalence in previous HF studies (i.e. the metabolic cluster in Tromp et al. (2018) or the common cluster in Lee et al. (2014) (Figure S2).

Each model was estimated with 100 replications. There were no cases with missing data. After establishing the optimal number of clusters, partial probabilities of being assigned to each cluster were calculated per patient (Figure S2). Thus, the identified clusters represented probabilistic groups of patients with similar combinations of comorbidities. Final group selection was done according to patients’ highest probability of being assigned to a group (Table S4).

In order to assess whether adding “hypertension” to the latent class model would increase model fit, we ran a 5 class LCA with this variable included. The BIC penalizes on the number of parameters in the model therefore it was used to compare the “hypertension” model with the main model:

|  | Main LCA model | Hypertension LCA model |
| --- | --- | --- |
| BIC | 4268117 | 4374723 |
| sBIC | 4267914 | 4374504 |
| LCA, latent class mode; BIC, Bayesian Information Criterion; sBIC, sample adjusted BIC | | |

As expected, adding “hypertension” to the set of comorbidities used to derive the clusters did not improve model fit as the BIC for this model was higher compared to the main model.

**Descriptive statistics**

Baseline characteristics were described according to each comorbidity cluster using numbers and percentages for categorical data and means and standard deviations and medians and interquartile ranges for normally and non-normally distributed continuous variables.

**Negative binomial regressions**

The negative binomial distribution assumes that each patient has recurrent events according to an individual-specific Poisson event rate and that those vary according to a gamma distribution.

**HF-specific admission and mortality analyses**

The associations between cluster and HF-specific admission and mortality were modelled using time-dependent coefficients.

**Sensitivity analysis: pseudo-class draws**

We used latent class variables to assess association with outcomes. Usual practice is to assign observations (i.e. patients) to one of the latent classes (clusters) based on the maximum posterior probabilities. The assigned class membership is thus treated as an observed variable, however this method ignores the uncertainty of being in each cluster, for each patient.

Due to the uncertainty in predicted class membership, we employed a multiple imputation (pseudo-class) approach, in a sensitivity analysis, in order to account for any uncertainty that comorbidity cluster classification would add to the outcome statistical analysis. “Pseudo-class draws” is a method to reduce the errors introduced by ignoring the probability of each observation being assigned to classes other than “highest probability” one considered in our main approach. With the pseudo-class approach, we make multiple random draws from the posterior probability distributions of observations (in our case, each patient has 5 probabilities of belonging to each of the clusters we identified). The random draws are used as multiple imputations of each observation’s class membership as if the class membership were missing. We used 20 random draws from a uniform distribution to generate 20 simulated class memberships for each patient (given their original partial class probabilities).

Using each imputed class membership as the exposure to test the association between class membership (comorbidity cluster) and outcome (time-to-admission), we fit 20 Cox regression models, adjusting for the same covariates used in the main outcome analysis. Estimates from the 20 models fitted to imputed datasets were combined using Rubin’s rule. This allows for the standard error of the association between comorbidity cluster and time to admission to be calculated. Results from the imputation analysis were attenuated, but similar to the main analysis (Table S9).


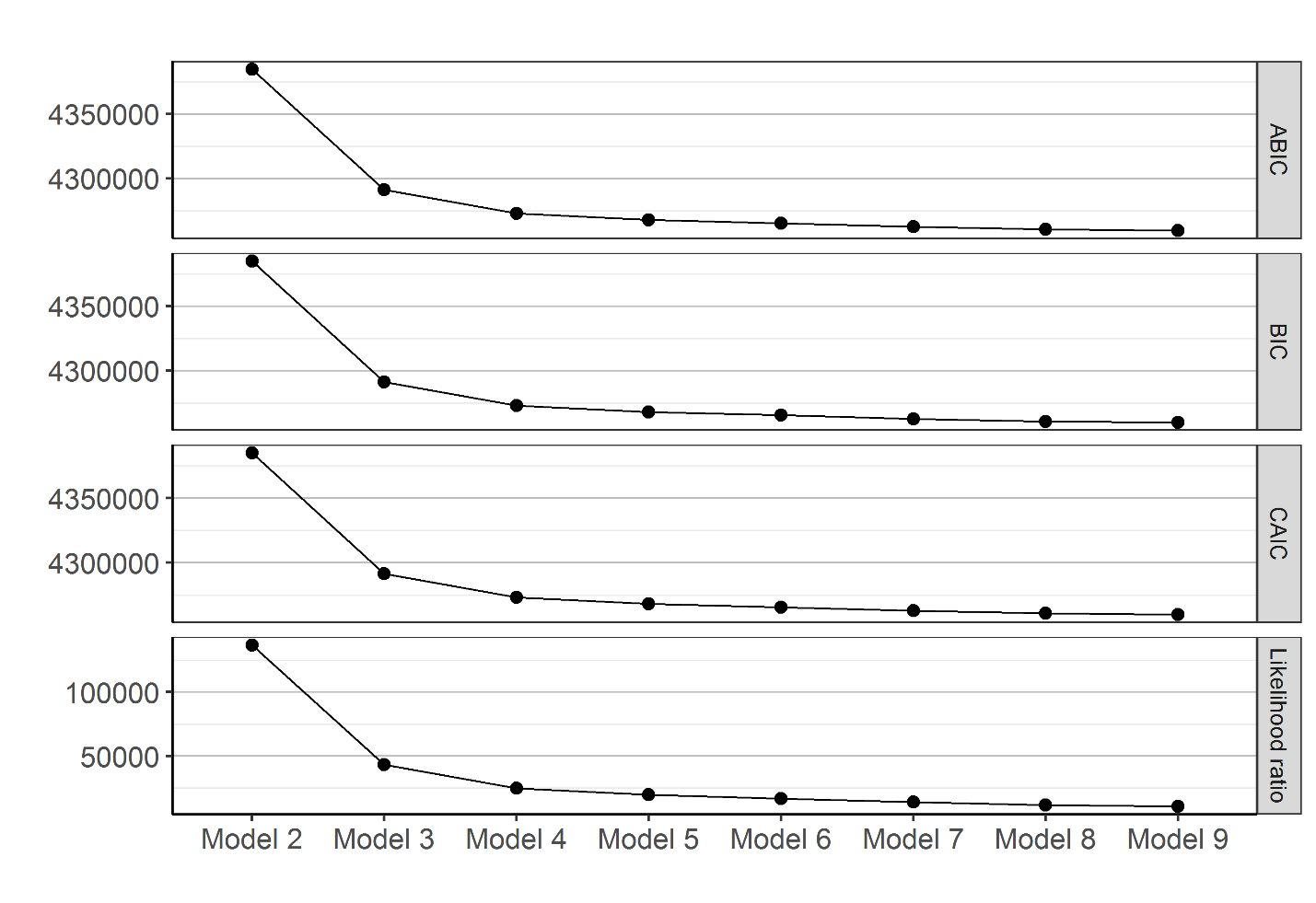


**Figure S1**. Fit indices for the 2 to 9 class solution models derived using latent class analysis


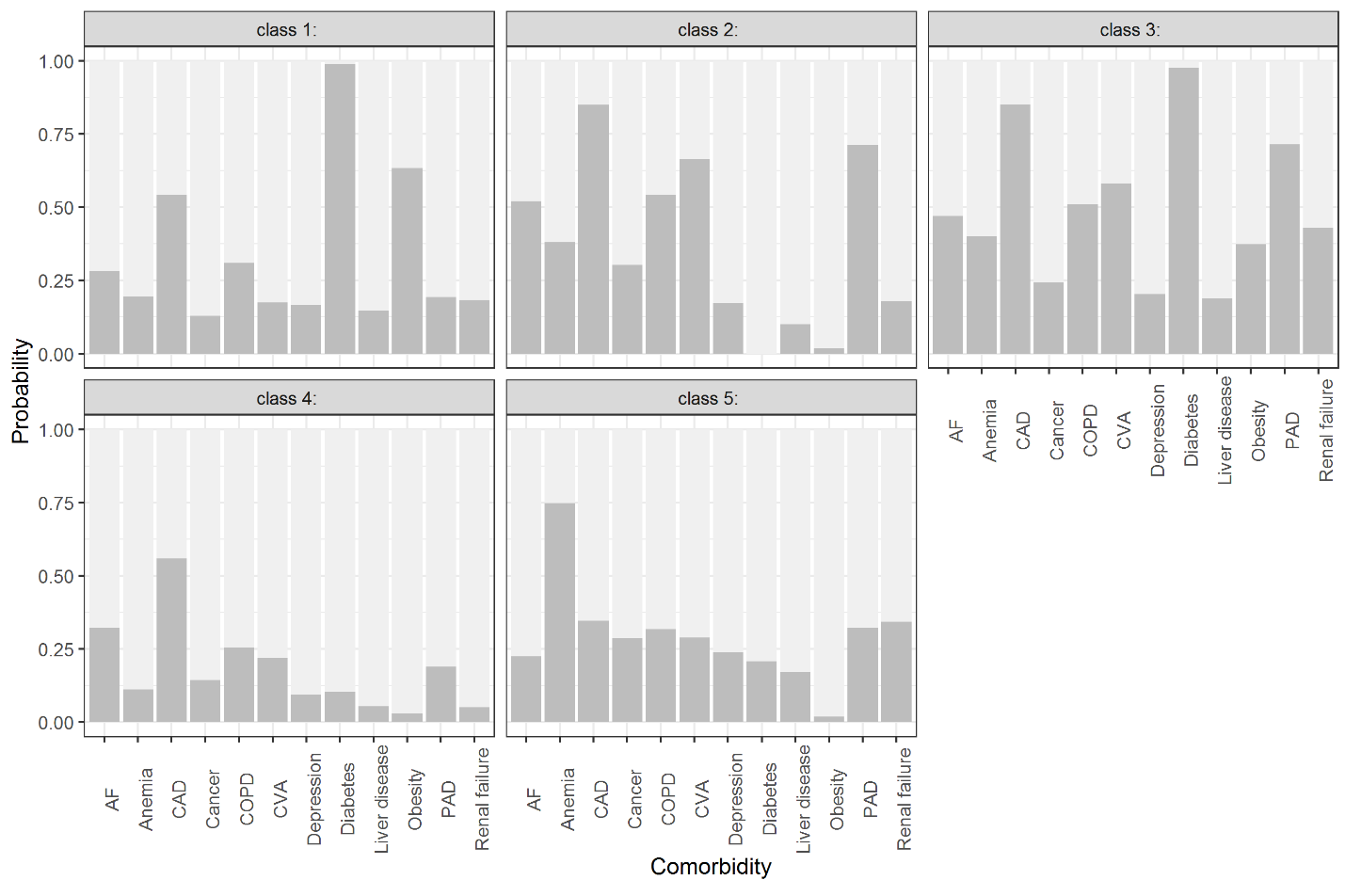


**Figure S2.** Partial probabilities of class membership for all variables used in deriving the clusters, per class


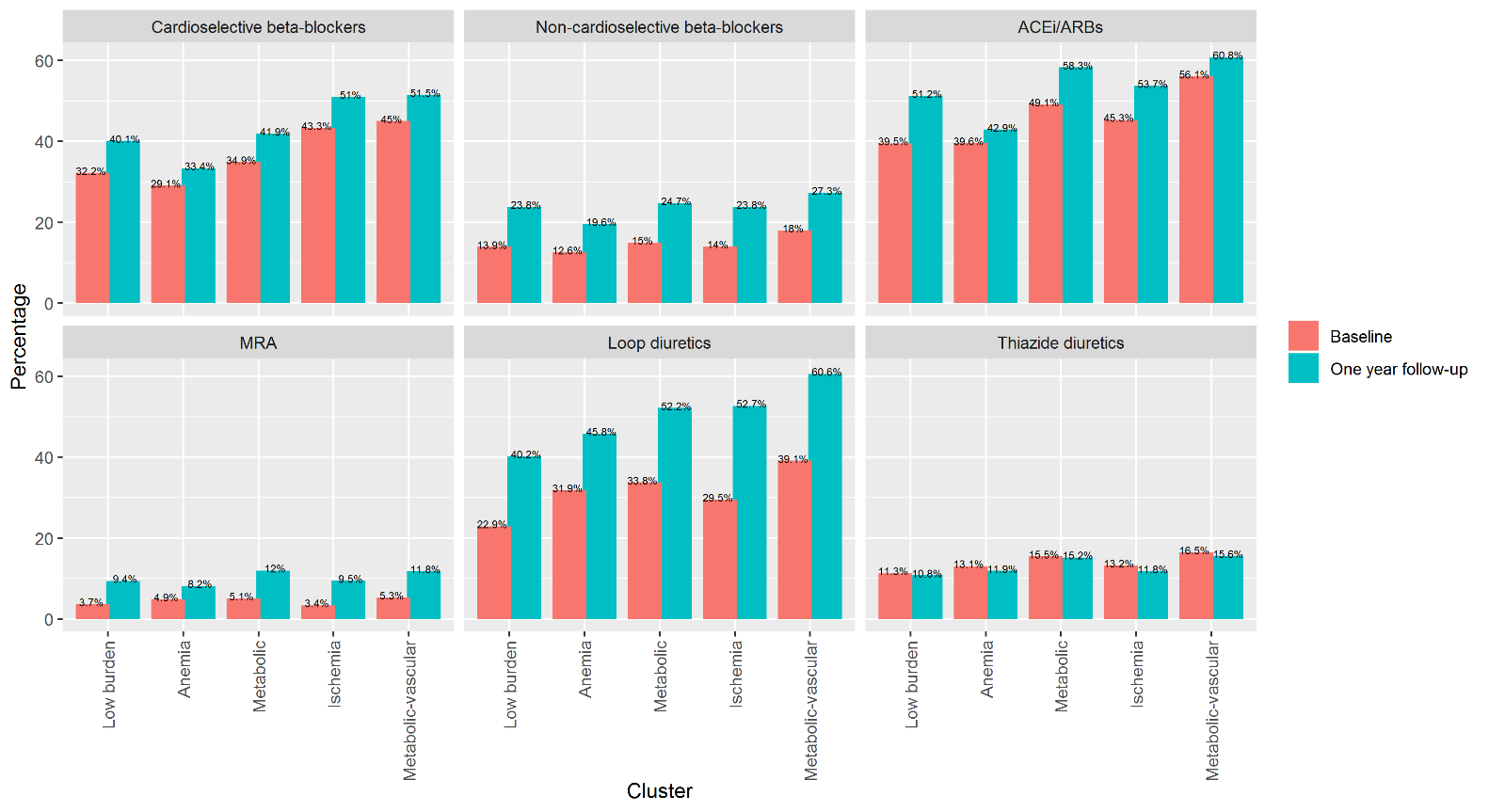


**Figure S3.** Prescription patterns for heart failure recommended medications as well as diuretics, from baseline to one-year follow-up, across comorbidity clusters (potassium-sparing not shown due to negligible proportions).

**
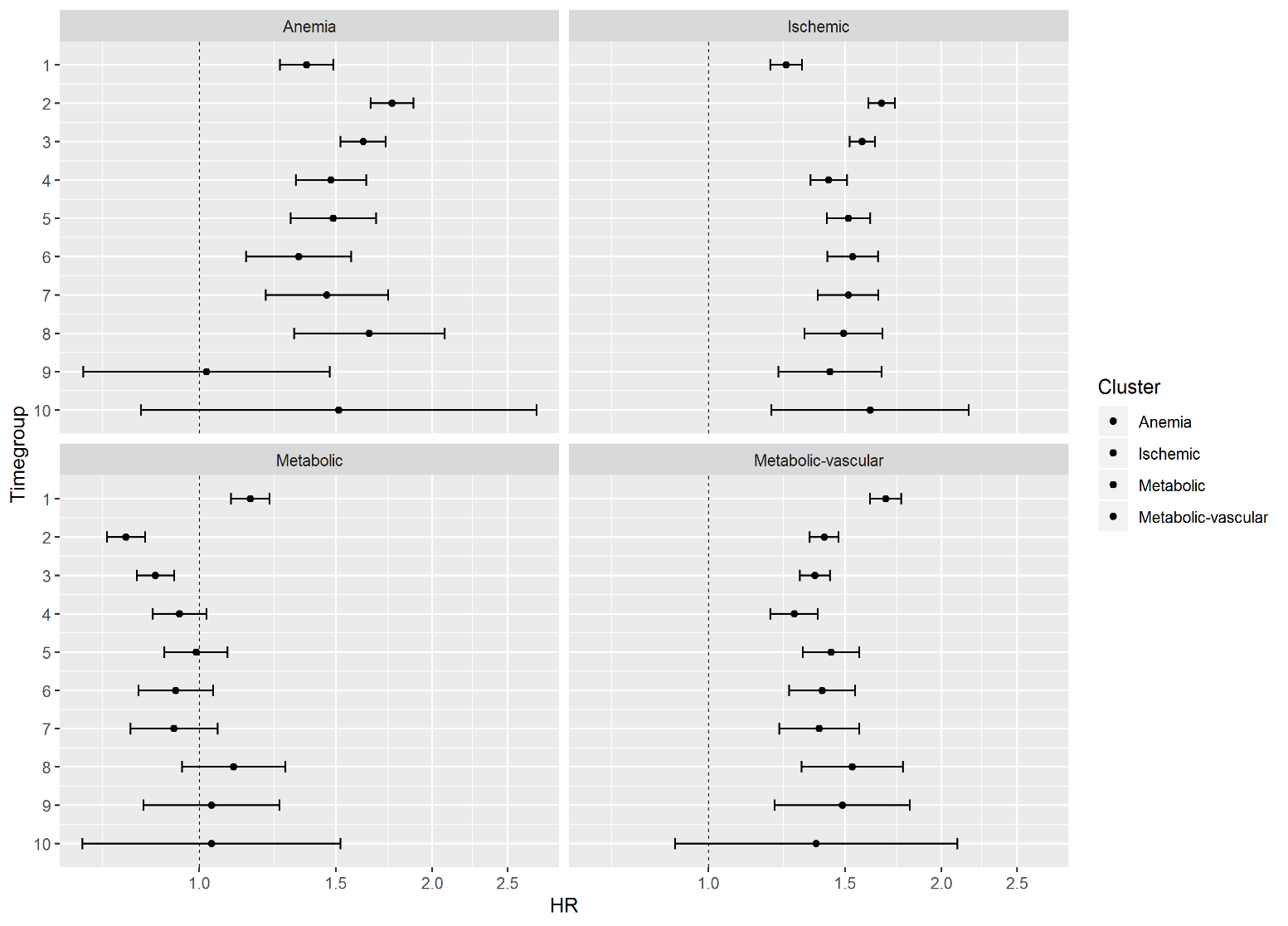
**

**Figure S4.** Hazard ratios (95%CI) for mortality per time group, according to comorbidity cluster

To explore the effect of the interaction, we used a step function for estimating the variation with time over one-year intervals and obtained stratified HRs per each time group. We observed that in the first 12 months of follow up (time group 1), the risk of death associated with all clusters was higher compared to the low-burden group. The association between the anemic, ischemic and metabolic-vascular clusters with risk of death, remained positive though lost significance in the last two periods for the anemic cluster, respectively last period for the metabolic-vascular cluster*.* Notably, the metabolic group was the only cluster that was associated with a decrease in risk of death – but only during time groups 2 and 3 (from 12 to 36 months).

**Table S1.** List of ICD9 and ICD10 codes used to identify heart failure patients

| **ICD-9 code** | **Diagnosis** |
| --- | --- |
| 42830 | Diastolic heart failure |
| 42831 | Acute diastolic heart failure |
| 42832 | Chronic diastolic heart failure |
| 42833 | Acute on chronic systolic heart failure |
| 42820 | Systolic heart failure, unspecified |
| 42821 | Acute systolic heart failure |
| 42822 | Chronic systolic heart failure |
| 42823 | Acute on chronic systolic heart failure |
| 42840 | Combined systolic and diastolic heart failure, unspecified |
| 42841 | Acute combined systolic and diastolic heart failure |
| 42842 | Chronic combined systolic and diastolic heart failure |
| 42843 | Acute on chronic combined systolic and diastolic heart failure |
| 4289 | Heart failure, unspecified |
| 4280 | Congestive heart failure, unspecified |
| 4281 | Left heart failure |
| **ICD10 code** | **Diagnosis** |
| I5020 | Unspecified systolic (congestive) heart failure |
| I5021 | Acute systolic (congestive) heart failure |
| I5022 | Chronic systolic (congestive) heart failure |
| I5023 | Acute on chronic systolic (congestive) heart failure |
| I503 | Diastolic (congestive) heart failure |
| I5030 | Unspecified diastolic (congestive) heart failure |
| I5031 | Acute diastolic (congestive) heart failure |
| I5032 | Chronic diastolic (congestive) heart failure |
| I5033 | Acute on chronic diastolic (congestive) heart failure |
| I5040 | Unspecified combined systolic (congestive) and diastolic (congestive) heart failure |
| I5041 | Acute combined systolic (congestive) and diastolic (congestive) heart failure |
| I5042 | Chronic combined systolic (congestive) and diastolic (congestive) heart failure |
| I5043 | Acute on chronic combined systolic (congestive) and diastolic (congestive) heart failure |
| I5084 | End stage heart failure |
| I5089 | Other heart failure |
| I500 | Heart failure, unspecified |
| I501 | Left ventricular failure |
| I11 | Hypertensive heart disease with congestive heart failure |

**Table S2.** Medication classes captured from pharmacy claims

| **Cardioselective beta-blockers** | **Noncardioselective beta-blockers** | **ACEi/ARBs** | **MRA** | **Loop diuretics** | **Thiazide diuretics** | **Potassium-sparing diuretics** |
| --- | --- | --- | --- | --- | --- | --- |
| acebutolol, atenolol,  betaxolol,  bisoprolol,  betoprolol,  nebivolol | carvedilol,  nadolol, labetalol, penbutolol, pindolol  propranolol | captopril, enalapril, lisinopril, ramipril, trandolapril, fosinopril, perindopril or candesartan, valsartan, losartan, telmisartan, irbesartan, quinapril | eplerencone, spironolactone | furosemide, bumetanide, torasemide | bendroflumethiazide, hydrochlorothiazide, metolazone, indapamide | amiloride, triamterene |

**Table S3**. Fit statistics for 2-9 latent class models

| Model | Observations | Log-likelihood | Log-likelihood ratio | No. estimated parameters | AIC | BIC | ABIC | CAIC | Entropy |
| --- | --- | --- | --- | --- | --- | --- | --- | --- | --- |
| Two-clusters | 318,384 | -2197053 | 136911.79 | 4070 | 4394156 | 4394423 | 4384816 | 4384920 | 0.45 |
| Three-clusters | 318,384 | -2149991 | 43181.79 | 4057 | 4300058 | 4300463 | 4291209 | 4291368 | 0.63 |
| Four-clusters | 318,384 | -2140743 | 24774.96 | 4044 | 4281589 | 4282133 | 4272926 | 4273139 | 0.69 |
| Five-clusters | 318,384 | -2133653 | 19639.76 | 4031 | 4267434 | 4268117 | 4267914 | 4268181 | 0.64 |
| Six-clusters | 318,384 | -2131796 | 16877.38 | 4018 | 4263746 | 4264568 | 4265275 | 4265597 | 0.6 |
| Seven-clusters | 318,384 | -2135271 | 13978.96 | 4005 | 4270723 | 4271683 | 4262500 | 4262876 | 0.57 |
| Eight-clusters | 318,384 | -2134134 | 11772.22 | 3992 | 4268473 | 4269573 | 4260417 | 4260847 | 0.55 |
| Nine-clusters | 318,384 | -2133552 | 10707.06 | 3979 | 4267336 | 4268574 | 4259475 | 4259959 | 0.52 |

**Table S4.**  Median (IQR) probability of group membership for the 5-class solution

| **Cluster** | **Median (IQR)** |
| --- | --- |
| Low-burden | 0.84 (0.67, 0.93) |
| Metabolic-vascular | 0.85 (0.67, 0.95) |
| Ischemic | 0.81 (0.64, 0.92) |
| Anemia | 0.58 (0.47, 0.7) |
| Metabolic | 0.70 (0.55, 0.90) |

**Table S5.** Baseline characteristics according to ejection fraction group, in patients with data available (N=13560)

|  | **HFrEF (n=3100)** | **HFmEF (n=1559)** | **HFpEF (n=8901)** | ***P - V*alue^a^** |
| --- | --- | --- | --- | --- |
| Age |  |  |  |  |
| Median [Q1, Q3] | 71.0 [61.0, 78.0] | 72.0 [63.0, 79.0] | 74.0 [66.0, 81.0] | <0.001 |
| **Sex** |  |  |  |  |
| Female | 1081 (34.9%) | 497 (31.9%) | 4815 (54.1%) | HFrEF vs HFpEF  HFmEF vs HFpEF  <0.001 |
| Male | 2019 (65.1%) | 1062 (68.1%) | 4086 (45.9%) |  |
| **Comorbidity cluster** |  |  |  | <0.01 |
| Low burden | 981 (31.6%) | 421 (27.0%) | 1728 (19.4%) |  |
| Metabolic-vascular | 610 (19.7%) | 375 (24.1%) | 2597 (29.2%) |  |
| Ischemic | 775 (25.0%) | 418 (26.8%) | 2459 (27.6%) |  |
| Anemia | 60 (1.9%) | 31 (2.0%) | 325 (3.7%) |  |
| Metabolic | 674 (21.7%) | 314 (20.1%) | 1792 (20.1%) |  |
| **Comorbidities at baseline** |  |  |  |  |
| COPD | 1014 (32.7%) | 562 (36.0%) | 3843 (43.2%) | HFrEF vs. HFpEF <0.001 |
| AF | 1326 (42.8%) | 736 (47.2%) | 4323 (48.6%) | HFrEF vs. HFpEF <0.001 |
| Alcohol misuse disorder | 105 (3.4%) | 57 (3.7%) | 304 (3.4%) | NS |
| Anemia | 606 (19.5%) | 350 (22.5%) | 2966 (33.3%) | HFrEF vs HFpEF  HFmEF vs HFpEF  <0.001 |
| CAD | 2586 (83.4%) | 1285 (82.4%) | 6360 (71.5%) | HFrEF vs HFpEF  HFmEF vs HFpEF  <0.001 |
| CVA | 1057 (34.1%) | 609 (39.1%) | 4085 (45.9%) | HFrEF vs HFmEF <0.01  HFrEF vs HFpEF  HFmEF vs HFpEF  <0.001 |
| Liver disease | 295 (9.5%) | 197 (12.6%) | 1306 (14.7%) | HFrEF vs HFmEF <0.01  HFrEF vs HFpEF  <0.001 |
| Cancer | 617 (19.9%) | 348 (22.3%) | 2018 (22.7%) | HFrEF vs HFpEF  <0.05 |
| Dementia | 128 (4.1%) | 54 (3.5%) | 574 (6.4%) | HFrEF vs HFpEF  HFmEF vs HFpEF  <0.001 |
| Depression | 400 (12.9%) | 224 (14.4%) | 1756 (19.7%) | HFrEF vs HFpEF  HFmEF vs HFpEF  <0.001 |
| Diabetes | 1278 (41.2%) | 685 (43.9%) | 4380 (49.2%) | HFrEF vs HFpEF  HFmEF vs HFpEF  <0.001 |
| PAD | 1280 (41.3%) | 715 (45.9%) | 4690 (52.7%) | HFrEF vs HFmEF <0.05  HFrEF vs HFpEF  HFmEF vs HFpEF  <0.001 |
| Hypertension | 2922 (94.3%) | 1480 (94.9%) | 8540 (95.9%) | HFrEF vs HFpEF  <0.001 |
| Renal failure | 576 (18.6%) | 357 (22.9%) | 2561 (28.8%) | HFrEF vs HFmEF <0.05  HFrEF vs HFpEF  HFmEF vs HFpEF  <0.001 |
| Peptic ulcer | 135 (4.4%) | 84 (5.4%) | 631 (7.1%) | HFrEF vs HFpEF  <0.001 |
| Obesity | 1231 (39.7%) | 624 (40.0%) | 4114 (46.2%) | HFrEF vs HFmEF <0.01  HFrEF vs HFpEF  HFmEF vs HFpEF  <0.001 |
| **Place of diagnosis** |  |  |  | HFrEF vs HFpEF  HFmEF vs HFpEF  <0.001 |
| Outpatient | 1509 (48.7%) | 784 (50.3%) | 3649 (41.0%) |  |
| Inpatient | 1591 (51.3%) | 775 (49.7%) | 5252 (59.0%) |  |
| **Insurance provider** |  |  |  | HFrEF vs HFmEF <0.01  HFrEF vs HFpEF  HFmEF vs HFpEF  <0.001 |
| Medicare Advantage | 2065 (66.6%) | 1113 (71.4%) | 6843 (76.9%) |  |
| Commercial | 1035 (33.4%) | 446 (28.6%) | 2058 (23.1%) |  |
| **Education** |  |  |  | NS |
| High School Diploma | 930 (30.0%) | 436 (28.0%) | 2565 (28.8%) |  |
| Less than Bachelor Degree | 1767 (57.0%) | 882 (56.6%) | 5058 (56.8%) |  |
| Bachelor Degree Plus | 392 (12.6%) | > 233 * | 1225 (13.8%) |  |
| Missing | 11 (0.3%) | <11 * | 53 (0.5%) |  |
| **Income** |  |  |  | HFrEF vs HFpEF  HFmEF vs HFpEF  <0.001 |
| <$40,000 | 841 (27.1%) | 387 (24.8%) | 2794 (31.4%) |  |
| $40,000-$74,000 | 888 (28.6%) | 441 (28.3%) | 2463 (27.7%) |  |
| $75,000- $124,999 | 737 (23.8%) | 381 (24.4%) | 1777 (20.0%) |  |
| $125,000- $199,999 | 224 (7.2%) | 131 (8.4%) | 574 (6.4%) |  |
| $200,000+ | 108 (3.5%) | 60 (3.8%) | 266 (3.0%) |  |
| Missing | 302 (9.7%) | 159 (10.2%) | 1027 (11.5%) |  |
| **Race** |  |  |  | NS |
| White | 2408 (77.7%) | 1251 (80.2%) | 6937 (77.9%) |  |
| Asian | 40 (1.3%) | 15 (1.0%) | 92 (1.0%) |  |
| Black | 363 (11.7%) | 155 (9.9%) | 1048 (11.8%) |  |
| Hispanic | 97 (3.1%) | 56 (3.6%) | 291 (3.3%) |  |
| Missing | 192 (6.2%) | 82 (5.3%) | 533 (6.0%) |  |
| **Smoking status** |  |  |  | HFrEF vs HFpEF <0.001  HFmEF vs HFpEF <0.05 |
| Current smoker | 566 (18.3%) | 245 (15.7%) | 1196 (13.4%) |  |
| Never smoked | 807 (26.0%) | 424 (27.2%) | 2729 (30.7%) |  |
| Not currently smoking | 357 (11.5%) | 174 (11.2%) | 1017 (11.4%) |  |
| Previously smoked | 1021 (32.9%) | 569 (36.5%) | 2992 (33.6%) |  |
| Missing | 349 (11.3%) | 147 (9.4%) | 967 (10.9%) |  |
| **Medication at baseline** |  |  |  |  |
| ACEis/ ARBs | 1796 (57.9%) | 868 (55.7%) | 4434 (49.8%) | HFrEF vs HFpEF  HFmEF vs HFpEF  <0.001 |
| Cardioselective beta-blockers | 1187 (38.3%) | 767 (49.2%) | 4241 (47.6%) | HFrEF vs HFmEF  HFrEF vs HFpEF  <0.001 |
| Non-cardioselective beta-blockers | 920 (29.7%) | 328 (21.0%) | 1247 (14.0%) | HFrEF vs HFmEF <0.01  HFrEF vs HFpEF  HFmEF vs HFpEF  <0.001 |
| MRA | 230 (7.4%) | 80 (5.1%) | 425 (4.8%) | HFrEF vs HFmEF <0.05  HFrEF vs HFpEF <0.001 |
| Thiazide diuretics | 307 (9.9%) | 186 (11.9%) | 1373 (15.4%) | HFrEF vs HFpEF <0.001  HFmEF vs HFpEF  <0.01 |
| Loop diuretics | 800 (25.8%) | 388 (24.9%) | 3209 (36.1%) | HFrEF vs HFpEF  HFmEF vs HFpEF  <0.001 |
| Double therapy (ACEi/ARB and beta-blocker) | 1391 (44.9%) | 686 (44.0%) | 2978 (33.5%) | HFrEF vs HFpEF  HFmEF vs HFpEF  <0.001 |
| Triple therapy (ACEi/ARB, MRA and beta-blocker) | 168 (5.4%) | 50 (3.2%) | 191 (2.1%) | HFrEF vs HFmEF <0.01  HFrEF vs HFpEF <0.001  HFmEF vs HFpEF  <0.05 |
| ^a^  Bonferroni adjusted, significant comparisons shown; NS= non-significant  * Exact numbers not presented in order to comply with Centres for Medicare and Medicaid Services cell size suppression policy  Abbreviations: IQR, inter-quartile range; NS, non-significant; AF, atrial fibrillation; CAD, coronary artery disease; CVA, cerebrovascular disease; PAD, peripheral artery disease; COPD, chronic obstructive pulmonary disease; US, United States, ACEis, angiotensin-converting-enzyme inhibitors; ARB, angiotensin receptor blockers; MRA, mineralocorticoid receptor antagonist; HFrEF, heart failure with reduced ejection fraction; HFmEF, heart failure with mid-range ejection fraction; HFpEF, heart failure with preserved ejection fraction | | | | |

More patients with HFpEF were diagnosed outpatient compared to the other two groups and were more likely to be on Medicare rather than commercial insurance plans. Education, race and income were similar across groups. There was a higher prevalence of current smokers in patients with HFrEF. Regarding medication, those with HFrEF had the highest rates of ACEi/ARBs prescriptions, similar to those with HFmEF. Cardioselective beta-blocker prescription was highest in HFmEF and HFpEF, while prescription for non-selective beta-blockers was less common, compared to HFrEF. Percentages of double therapy were highest in the HFrEF and HFmEF groups, while triple therapy was the least often encountered treatment regimen across all three groups.

**Table S6.** Distribution of ejection fraction group and smoking status across comorbidity clusters in patients with data available

| **Comorbidity Cluster** | **Low burden** | **Metabolic-vascular** | **Ischemic** | **Anemia** | **Metabolic** | **Overall** | ***P* Value** |
| --- | --- | --- | --- | --- | --- | --- | --- |
| **Smoking status (n=35,721)** |  |  |  |  |  |  | ^a^ |
| Current smoker | 1350 (1.6%) | 1496 (2%) | 1797 (2.2%) | 200 (1.3%) | 1145 (1.8%) | 5988(1.9%) |  |
| Never smoked | 3011 (3.5%) | 3089 (4.2%) | 2647 (3.2%) | 565 (3.8%) | 3205 (5.1%) | 12,517 (3.9%) |  |
| Not currently smoking | 1131 (1.4%) | 924 (1.3%) | 1063 (1.3%) | 177 (1.2%) | 908 (1.4%) | 4203 (1.3%) |  |
| Previously smoked | 2418 (2.9%) | 3882 (5.3%) | 3484 (4.2%) | 474 (3.2%) | 2755 (4.4%) | 13,013 (4.1%) |  |
| Missing | 75,667 (90.5%) | 63,893 (87.2%) | 74,292 (89.2%) | 13,543 (90.5%) | 55,268 (87.3%) | 282,663 (88.8%) |  |
| **Ejection fraction**  **(n=13,560)** |  |  |  |  |  |  | ^b^ |
| HFrEF | 981 (1.2%) | 610 (0.8%) | 775 (0.9%) | 60 (0.4%) | 674 (1.1%) | 3100 (1.0%) |  |
| HFmEF | 421 (0.5%) | 375 (0.5%) | 418 (0.5%) | 31 (0.2%) | 314 (0.5%) | 1559 (0.5%) |  |
| HFpEF | 1728 (2.1%) | 2597 (3.5%) | 2459 (3.0%) | 325 (2.2%) | 1792 (2.8%) | 8901 (2.8%) |  |
| Missing | 80,447 (96.3%) | 69,702 (95.1%) | 79,631 (95.6%) | 14,543 (97.2%) | 60,501 (95.6%) | 304,824 (95.7%) |  |
| Abbreviations: HFmEF, heart failure with mid-range ejection fraction [EF] [40 %<EF<50%]; HFpEF, heart failure with preserved ejection fraction HFpEF [EF≥50%]; HFrEF, heart failure with reduced ejection fraction [EF≤40%]  ^a^  Non-significant pairwise Bonferroni adjusted comparisons: anemia vs. metabolic  ^b^ Non-significant pairwise Bonferroni adjusted comparisons: metabolic-vascular vs. anemia; anemia vs. metabolic; ischemic vs. metabolic | | | | | | | |

Ejection fraction group data was available only in 13,560 patients and smoking status in 35,721. With the caveat of considerable missing data on these variables, we observed the highest prevalence of HFpEF in the metabolic-vascular cluster, whilst the prevalence of HFrEF appeared to be relatively uniform with the exception of the anemic subgroup, where prevalence was lower compared to all other clusters (although this comparison may be limited by the relatively small number of patients in this group). Smoking prevalence did not appear to vary according to cluster, however there were slightly more patients that had smoked previously in the metabolic-vascular group, compared to the other groups.

**Table S7.** Frequency of admission to hospital across comorbidity clusters, within one-year follow-up

|  | **Low- burden** (n=83577) | **Metabolic-vascular** (n=73284) | **Ischemic** (n=83,283) | **Anemia**  (n=14959) | **Metabolic** (n=63281) | **Overall** (n=318384) | ***P* Value ^a^** |
| --- | --- | --- | --- | --- | --- | --- | --- |
| All-cause admission | 20,990 (25.1%) | 37,472 (51.1%) | 39,645 (47.6%) | 5637 (37.7%) | 19,473 (30.8%) | 123,217 (38.7%) | <0.001 |
| HF-specific admission | 4594 (5.5%) | 8921 (12.2%) | 9384 (11.3%) | 915 (6.1%) | 4352 (6.9%) | 28,166 (8.8%) | <0.001 |
| ^a^ Bonferroni adjusted | | | | | | | |

**Table S8.** Association between all-cause admission to hospital and comorbidity clusters, adjusted for ejection fraction and smoking status, within one-year follow-up (N=11294)

| \|  \| **Univariable HR (95% CI)** \| **Multivariable HR (95% CI)** \| \| --- \| --- \| --- \| \| Age (years) \| 1.01 (1.00-1.01, p<0.001) \| 0.99 (0.99-1.00, p<0.001) \| \| Sex (Male vs. Female) \| 0.98 (0.97-0.99, p<0.001) \| 0.86 (0.81-0.91, p<0.001) \| \| Dementia \| 1.25 (1.23-1.28, p<0.001) \| 1.32 (1.18-1.48, p<0.001) \| \| Peptic ulcer \| 1.65 (1.62-1.69, p<0.001) \| 1.53 (1.39-1.70, p<0.001) \| \| Alcohol misuse disorder \| 1.32 (1.28-1.36, p<0.001) \| 1.43 (1.24-1.64, p<0.001) \| \| Hypertension \| 2.06 (1.99-2.14, p<0.001) \| 1.66 (1.37-2.01, p<0.001) \| \| **Race (ref: White)** \|  \|  \| \| Black \| 1.06 (1.04-1.07, p<0.001) \| 1.07 (0.98-1.17, p=0.118) \| \| Hispanic \| 0.93 (0.91-0.95, p<0.001) \| 0.89 (0.75-1.05, p=0.162) \| \| Asian \| 0.83 (0.79-0.86, p<0.001) \| 1.01 (0.77-1.32, p=0.958) \| \| **Education (ref: Bachelor Degree Plus)** \|  \|  \| \| High School Diploma \| 1.15 (1.12-1.17, p<0.001) \| 1.05 (0.95-1.16, p=0.363) \| \| Less than 12 grade \| 1.08 (0.98-1.19, p=0.133) \| 1.81 (0.58-5.65, p=0.307) \| \| Less than Bachelor Degree \| 1.08 (1.06-1.10, p<0.001) \| 1.02 (0.93-1.11, p=0.719) \| \| Commercial vs Medicare Advantage \| 0.81 (0.80-0.82, p<0.001) \| 0.88 (0.81-0.96, p=0.005) \| \| Inpatient vs Outpatient \| 1.32 (1.31-1.34, p<0.001) \| 1.14 (1.07-1.21, p<0.001) \| \| **Medications at baseline** \|  \|  \| \| Cardioselective beta-blockers \| 1.07 (1.06-1.08, p<0.001) \| 0.95 (0.89-1.01, p=0.093) \| \| Non-cardioselective beta-blockers \| 1.03 (1.01-1.04, p=0.001) \| 0.97 (0.89-1.05, p=0.451) \| \| ACEIs/ARBs \| 1.06 (1.05-1.07, p<0.001) \| 0.96 (0.91-1.02, p=0.216) \| \| MRA \| 1.03 (1.00-1.06, p=0.036) \| 0.95 (0.83-1.08, p=0.408) \| \| Thiazide diuretics \| 1.07 (1.05-1.08, p<0.001) \| 1.04 (0.95-1.13, p=0.396) \| \| Potassium-sparing diuretics \| 1.35 (1.15-1.59, p<0.001) \| 1.10 (0.52-2.31, p=0.806) \| \| Loop diuretics \| 1.21 (1.20-1.23, p<0.001) \| 1.16 (1.08-1.23, p<0.001) \| \| **Cluster (ref: Low burden)** \|  \|  \| \| Metabolic-vascular \| 2.43 (2.39-2.47, p<0.001) \| 2.11 (1.92-2.32, p<0.001) \| \| Ischemic \| 2.20 (2.17-2.24, p<0.001) \| 1.90 (1.72-2.08, p<0.001) \| \| Anemia \| 1.62 (1.58-1.67, p<0.001) \| 1.70 (1.43-2.03, p<0.001) \| \| Metabolic \| 1.26 (1.24-1.29, p<0.001) \| 1.17 (1.05-1.30, p=0.004) \| \| **Smoking status (ref: Current smoker)** \| - \| - \| \| Never smoked \| 0.80 (0.76-0.84, p<0.001) \| 0.87 (0.80-0.95, p=0.003) \| \| Not currently smoking \| 0.88 (0.83-0.93, p<0.001) \| 0.94 (0.84-1.04, p=0.238) \| \| Previously smoked \| 0.85 (0.81-0.89, p<0.001) \| 0.87 (0.80-0.95, p=0.001) \| \| LVEF \| 1.00 (1.00-1.00, p=0.015) \| 1.00 (1.00-1.00, p=0.817) \| \| Abbreviations: HR, hazard ratio; CI, confidence intervals; ref, reference; ACEis, angiotensin-converting-enzyme inhibitors; ARB, angiotensin receptor blockers; MRA, mineralocorticoid receptor antagonists; LVEF, left ventricular ejection fraction; ref, reference \| \| \| |
| --- | --- | --- | --- | --- | --- | --- | --- | --- | --- | --- | --- | --- | --- | --- | --- | --- | --- | --- | --- | --- | --- | --- | --- | --- | --- | --- | --- | --- | --- | --- | --- | --- | --- | --- | --- | --- | --- | --- | --- | --- | --- | --- | --- | --- | --- | --- | --- | --- | --- | --- | --- | --- | --- | --- | --- | --- | --- | --- | --- | --- | --- | --- | --- | --- | --- | --- | --- | --- | --- | --- | --- | --- | --- | --- | --- | --- | --- | --- | --- | --- | --- | --- | --- | --- | --- | --- | --- | --- | --- | --- | --- | --- | --- | --- | --- | --- | --- | --- | --- | --- | --- | --- | --- | --- | --- | --- | --- | --- |

EF was introduced as continuous term (LVEF) - interaction between LVEF and cluster not significant (model not shown).

**Table S9.** Adjusted association between admission to hospital and comorbidity cluster, within one-year follow-up. Results from 20 models using imputed class assignments (estimates combined using Rubin’s rule) and results from the main analysis

|  | **Imputation model** | | | | | **Main analysis** | | | | |
| --- | --- | --- | --- | --- | --- | --- | --- | --- | --- | --- |
|  | **Estimate** | **SE** | **Lower 95%CI** | **Upper 95%CI** | ***P* Value** | **Estimate** | **SE** | **Lower 95%CI** | **Upper 95%CI** | ***P* Value** |
| **Model covariate** |  |  |  |  |  |  |  |  |  |  |
| Age (years) | -0.006 | 0.0003 | -0.006 | -0.005 | <0.001 | -0.007 | 0.0003 | -0.008 | -0.007 | <0.001 |
| Sex (Male vs. Female) | -0.005 | 0.006 | -0.017 | 0.006 | 0.384 | -0.018 | 0.006 | -0.030 | -0.006 | <0.05 |
| Dementia | 0.168 | 0.010 | 0.147 | 0.189 | <0.001 | 0.141 | 0.010 | 0.120 | 0.162 | <0.001 |
| Peptic ulcer | 0.382 | 0.012 | 0.359 | 0.405 | <0.001 | 0.353 | 0.011 | 0.331 | 0.376 | <0.001 |
| Alcohol misuse disorder | 0.215 | 0.016 | 0.183 | 0.247 | <0.001 | 0.202 | 0.016 | 0.170 | 0.233 | <0.001 |
| Hypertension | 0.506 | 0.019 | 0.468 | 0.544 | <0.001 | 0.456 | 0.019 | 0.417 | 0.493 | <0.001 |
| **Race (ref: White)** |  |  |  |  |  |  |  |  |  |  |
| Black | 0.005 | 0.008 | -0.011 | 0.022 | 0.517 | 0.005 | 0.008 | -0.001 | 0.021 | 0.496 |
| Hispanic | -0.088 | 0.011 | -0.112 | -0.065 | <0.001 | -0.09 | 0.011 | -0.118 | -0.072 | <0.001 |
| Asian | -0.149 | 0.022 | -0.192 | -0.105 | <0.001 | -0.149 | 0.022 | -0.193 | -0.106 | <0.001 |
| **Education (ref: Bachelor Degree Plus)** |  |  |  |  |  |  |  |  |  |  |
| High School Diploma | 0.049 | 0.011 | 0.029 | 0.070 | <0.001 | 0.043 | 0.010 | 0.022 | 0.063 | <0.001 |
| Less than 12 grade | 0.050 | 0.053 | -0.053 | 0.154 | 0.343 | 0.041 | 0.052 | -0.060 | 0.144 | 0.424 |
| Less than Bachelor Degree | 0.016 | 0.009 | 0.003 | 0.036 | 0.09 | 0.014 | 0.009 | -0.005 | 0.033 | 0.153 |
| Business line (commercial vs. Medicare Advantage) | -0.140 | 0.008 | -0.155 | -0.124 | <0.001 | -0.130 | 0.007 | -0.145 | -0.114 | <0.001 |
| Place of diagnosis (Inpatient vs. outpatient) | 0.248 | 0.006 | 0.235 | 0.260 | <0.001 | 0.233 | 0.006 | 0.221 | 0.245 | <0.001 |
| **Medications at baseline** |  |  |  |  |  |  |  |  |  |  |
| Cardioselective beta- blockers | -0.009 | 0.006 | -0.022 | 0.003 | 0.130 | -0.026 | 0.006 | -0.038 | -0.013 | <0.001 |
| Noncardio-selective beta-blockers | 0.007 | 0.009 | -0.009 | 0.024 | =0.395 | -0.005 | 0.008 | -0.022 | 0.011 | 0.502 |
| ACEIs/ARBs | 0.008 | 0.006 | -0.020 | 0.003 | p=0.175 | -0.013 | 0.006 | -0.025 | -0.001 | <0.05 |
| MRA | -0.018 | 0.015 | -0.046 | 0.010 | P=0.215 | -0.016 | 0.145 | -0.044 | 0.012 | 0.265 |
| Thiazide diuretics | 0.029 | 0.008 | 0.013 | 0.047 |  | 0.030 | 0.008 | 0.013 | 0.046 | <0.001 |
| Potassium-sparing diuretics | 0.213 | 0.086 | 0.044 | 0.383 | <0.05 | 0.199 | 0.085 | 0.032 | 0.367 | <0.05 |
| Loop diuretics | 0.168 | 0.007 | 0.155 | 0.181 | <0.001 | 0.162 | 0.006 | 0.149 | 0.174 | <0.001 |
| **Cluster (ref: Low-burden)** |  |  |  |  |  |  |  |  |  |  |
| Metabolic-vascular | 0.657 | 0.011 | 0.638 | 0.680 | <0.001 | 0.793 | 0.010 | 0.775 | 0.811 | <0.001 |
| Ischemic | 0.581 | 0.012 | 0.556 | 0.604 | <0.001 | 0.731 | 0.009 | 0.713 | 0.749 | <0.001 |
| Anemia | 0.295 | 0.018 | 0.259 | 0.330 | <0.001 | 0.398 | 0.015 | 0.368 | 0.429 | <0.001 |
| Metabolic | 0.147 | 0.012 | 0.122 | 0.172 | <0.001 | 0.151 | 0.010 | 0.130 | 0.171 | <0.001 |
| Abbreviations: SE, standard error; CI, confidence interval; ref, reference; ACEi, angiotensin-converting-enzyme inhibitors; ARB, angiotensin receptor blockers; MRA, mineralocorticoid receptor antagonists; ref, reference. | | | | | | | | | | |

| **Table S10.** Association between heart failure-specific admission to hospital and comorbidity clusters, with time-varying coefficient due to non-proportional hazards, within one-year follow-up   \|  \| **Univariable HR (95% CI)** \| **Multivariable HR (95% CI)** \| \| --- \| --- \| --- \| \| Age (years) \| 1.01 (1.00-1.01, p<0.001) \| 1.01 (1.00-1.01, p<0.001) \| \| Male vs. Female \| 0.98 (0.97-0.99, p<0.001) \| 1.08 (1.06-1.11, p<0.001) \| \| Dementia \| 1.25 (1.23-1.28, p<0.001) \| 0.87 (0.83-0.91, p<0.001) \| \| Peptic ulcer \| 1.65 (1.62-1.69, p<0.001) \| 1.10 (1.05-1.16, p<0.001) \| \| Alcohol misuse disorder \| 1.32 (1.28-1.36, p<0.001) \| 0.86 (0.80-0.93, p<0.001) \| \| Hypertension \| 2.06 (1.99-2.14, p<0.001) \| 2.02 (1.83-2.22, p<0.001) \| \| **Race (ref: White)** \|  \|  \| \| Black \| 1.06 (1.04-1.07, p<0.001) \| 1.14 (1.11-1.18, p<0.001) \| \| Hispanic \| 0.93 (0.91-0.95, p<0.001) \| 1.02 (0.97-1.07, p=0.498) \| \| Asian \| 0.83 (0.79-0.86, p<0.001) \| 1.05 (0.86-1.14, p=0.255) \| \| **Education (ref: Bachelor Degree Plus)** \|  \|  \| \| High School Diploma \| 1.15 (1.12-1.17, p<0.001) \| 1.03 (0.99-1.08, p=0.134) \| \| Less than 12 grade \| 1.08 (0.98-1.19, p=0.133) \| 1.06 (0.86-1.30, p=0.596) \| \| Less than Bachelor Degree \| 1.08 (1.06-1.10, p<0.001) \| 1.01 (0.96-1.05, p=0.789) \| \| Business line (Commercial vs. Medicare Advantage) \| 0.81 (0.80-0.82, p<0.001) \| 1.02 (0.99-1.06, p=0.135) \| \| Place of diagnosis (inpatient vs outpatient) \| 1.32 (1.31-1.34, p<0.001) \| 1.23 (1.20-1.26, p<0.001) \| \| **Medication at baseline** \|  \|  \| \| Cardioselective beta-blockers \| 1.07 (1.06-1.08, p<0.001) \| 0.99 (1.00-1.01, p=0.372) \| \| Non-cardioselective-selective beta-blockers \| 1.03 (1.01-1.04, p=0.001) \| 1.15 (1.11-1.19, p<0.001) \| \| ACEIs/ARBs \| 1.06 (1.05-1.07, p<0.001) \| 1.02 (1.00 -1.05, p=0.151) \| \| MRA \| 1.03 (1.00-1.06, p=0.036) \| 0.94 (0.88 – 0.99, p<0.05) \| \| Thiazide diuretics \| 1.07 (1.05-1.08, p<0.001) \| 1.10 (1.06-1.14, p<0.001) \| \| Potassium-sparing diuretics \| 1.35 (1.15-1.59, p<0.001) \| 0.88 (0.59-1.30, p=0.525) \| \| Loop diuretics \| 1.21 (1.20-1.23, p<0.001) \| 1.37 (1.33-1.41, p<0.001) \| \| **Cluster (ref: Low-burden)** \|  \|  \| \| Metabolic-vascular \| 2.43 (2.39-2.47, p<0.001) \| 1.85 (1.78-1.92, p<0.001) \| \| Metabolic-vascular * time ^a^ \| 1.16 (1.13- 1.19, p<0.001) \| 1.16 (1.13-1.19, p<0.001) \| \| Ischemic \| 2.20 (2.17-2.24, p<0.001) \| 1.81 (1.75-1.88, p<0.001) \| \| Ischemic * time ^b^ \| 1.13 (1.10-1.15, p<0.001) \| 1.13 (1.10-1.15, p<0.001) \| \| Anemia \| 1.62 (1.58-1.67, p<0.001) \| 1.02 (0.94 -1.10, p=0.622) \| \| Anemia * time ^c^ \| 1.05 (1.00-1.10, p<0.05) \| 1.05 (1.00-1.10, p<0.05) \| \| Metabolic \| 1.16 (0.88-1.52, p=0.289) \| 1.14 (1.09 -1.20, p<0.001) \| \| Metabolic * time ^d^ \| 1.05 (1.02-1.08, p<0.001) \| 1.05 (1.03-1.08, p<0.001) \| \| Abbreviations: HR, hazard ratio; CI, confidence intervals; ref, reference; ACEi, angiotensin-converting-enzyme inhibitors; ARB, angiotensin receptor blockers; MRA, mineralocorticoid receptor antagonists  Coefficients for interaction with time  ^a^ 0.147 , p-value<0.001  ^b^ 0.130 , p-value <0.001  ^c^ 0.051 , p-value=0.034  ^d^ 0.050 , p-value<0.001 \| \| \|   **Table S11.** Association between all-cause admission to hospital and number of comorbidities, within one-year follow-up |
| --- | --- | --- | --- | --- | --- | --- | --- | --- | --- | --- | --- | --- | --- | --- | --- | --- | --- | --- | --- | --- | --- | --- | --- | --- | --- | --- | --- | --- | --- | --- | --- | --- | --- | --- | --- | --- | --- | --- | --- | --- | --- | --- | --- | --- | --- | --- | --- | --- | --- | --- | --- | --- | --- | --- | --- | --- | --- | --- | --- | --- | --- | --- | --- | --- | --- | --- | --- | --- | --- | --- | --- | --- | --- | --- | --- | --- | --- | --- | --- | --- | --- | --- | --- | --- | --- | --- | --- | --- | --- | --- | --- | --- | --- | --- | --- | --- | --- | --- | --- | --- | --- | --- | --- | --- | --- |
| \|  \| **Univariable HR (95% CI)** \| **Multivariable HR (95%CI)** \| \| --- \| --- \| --- \| \| Age (years) \| 1.01 (1.00-1.01, p<0.001) \| 0.99 (0.99-0.99, p<0.001) \| \| Sex (male vs. female) \| 0.98 (0.97-0.99, p<0.001) \| 0.99 (0.98-1.00, p=0.052) \| \| **Race (ref: White)** \|  \|  \| \| Black \| 1.06 (1.04-1.07, p<0.001) \| 1.02 (1.00-1.04, p=0.018) \| \| Hispanic \| 0.93 (0.91-0.95, p<0.001) \| 0.92 (0.90-0.94, p<0.001) \| \| Asian \| 0.83 (0.79-0.86, p<0.001) \| 0.91 (0.87-0.95, p<0.001) \| \| **Education (ref: Bachelor Degree Plus)** \| - \| - \| \| High School Diploma \| 1.15 (1.12-1.17, p<0.001) \| 1.03 (1.01-1.06, p=0.001) \| \| Less than 12 grade \| 1.08 (0.98-1.19, p=0.133) \| 1.03 (0.93-1.15, p=0.526) \| \| Less than Bachelor Degree \| 1.08 (1.06-1.10, p<0.001) \| 1.00 (0.98-1.02, p=0.856) \| \| Commercial vs Medicare Advantage \| 0.81 (0.80-0.82, p<0.001) \| 0.92 (0.90-0.93, p<0.001) \| \| Inpatient vs outpatient diagnosis \| 1.32 (1.31 - 1.34, p<0.001) \| 1.20 (1.19-1.11, p<0.001) \| \| **Medications at baseline** \|  \|  \| \| Cardioselective beta-blockers \| 1.07 (1.06-1.08, p<0.001) \| 0.96 (0.94-0.97, p<0.001) \| \| Nonselective beta-blockers \| 1.03 (1.01-1.04, p=0.001) \| 0.99 (0.97-1.01, p=0.225) \| \| ACEIs/ARBs \| 1.06 (1.05-1.07, p<0.001) \| 0.98 (0.97-0.99, p<0.001) \| \| MRA \| 1.03 (1.00-1.06, p=0.036) \| 0.96 (0.94-0.99, p=0.011) \| \| Thiazide diuretics \| 1.07 (1.05-1.08, p<0.001) \| 1.02 (1.00-1.03, p=0.040) \| \| Potassium-sparing diuretics \| 1.35 (1.15-1.59, p<0.001) \| 1.17 (0.99-1.39, p=0.061) \| \| Loop diuretics \| 1.21 (1.20-1.23, p<0.001) \| 1.11 (1.10-1.12, p<0.001) \| \| **Number of comorbidities (ref: 2 or less)** \|  \|  \| \| 3 or 4 \| 1.90 (1.85-1.96, p<0.001) \| 1.92 (1.86-1.98, p<0.001) \| \| 5 or 6 \| 3.08 (2.99-3.16, p<0.001) \| 3.09 (3.00-3.18, p<0.001) \| \| 7 or 8 \| 4.33 (4.20-4.45, p<0.001) \| 4.32 (4.19-4.46, p<0.001) \| \| over 9 \| 5.87 (5.68-6.06, p<0.001) \| 5.79 (5.60-5.99, p<0.001) \| \| Abbreviations: HR, hazard ratio; CI, confidence intervals; ref, reference; ACEi, angiotensin-converting-enzyme inhibitors; ARB, angiotensin receptor blocker; MRA, mineralocorticoid receptor antagonists \| \| \|   **Table S12.** Association between mortality and comorbidity cluster with interaction between cluster and time (median 30 months follow-up)   \|  \| **Univariable HR (95% CI)** \| **Multivariable HR (95% CI)** \| \| --- \| --- \| --- \| \| Age (years) \| 1.06 (1.06-1.06, p<0.001) \| 1.05 (1.04-1.05, p<0.001) \| \| Sex (Male vs Female) \| 0.98 (0.97-1.00, p=0.009) \| 1.16 (1.14-1.18, p<0.001) \| \| **Race (ref: White)** \|  \|  \| \| Asian \| 0.78 (0.75-0.83, p<0.001) \| 0.83 (0.78-0.87, p<0.001) \| \| Black \| 0.89 (0.87-0.91, p<0.001) \| 0.93 (0.91-0.95, p<0.001) \| \| Hispanic \| 0.72 (0.70-0.75, p<0.001) \| 0.75 (0.73-0.78, p<0.001) \| \| Dementia \| 2.71 (2.63-2.77, p<0.001) \| 1.81 (1.78-1.85, p<0.001) \| \| Peptic ulcer \| 1.34 (1.30-1.38, p<0.001) \| 1.12 (1.09-1.16, p=0.001) \| \| Alcohol misuse disorder \| 1.11 (1.07-1.15, p<0.001) \| 1.30 (1.25-1.36 , p<0.001) \| \| Hypertension \| 2.09 (2.01-2.19, p<0.001) \| 1.11 (1.06-1.16, p<0.001) \| \| **Education (ref: Bachelor Degree)** \|  \|  \| \| High School Diploma \| 1.09 (1.06-1.11, p<0.001) \| 1.13 (1.10-1.16, p<0.001) \| \| Less than 12 grade \| 0.88 (1.14-1.02, p=0.080) \| 1.17 (1.02-1.37, p<0.05) \| \| Less than Bachelor Degree \| 1.03 (1.01-1.06, p=0.003) \| 1.03 (1.01-1.06, p<0.01) \| \| Business line (Commercial vs. Medicare Advantage \| 0.45 (0.44-0.46, p<0.001) \| 0.81 (0.80-0.83, p<0.001) \| \| Place of diagnosis (inpatient vs outpatient) \| 1.19 (1.17-1.21, p<0.001) \| 1.24 (1.22-1.25, p<0.001) \| \| **Medications at baseline** \|  \|  \| \| Cardioselective beta-blockers \| 1.14 (1.13-1.16, p<0.001) \| 0.97 (0.96-0.98, p<0.001) \| \| Non-cardioselective beta-blockers \| 0.94 (0.92-0.96, p<0.001) \| 0.98 (0.96-0.99, p<0.05) \| \| ACEis/ARBs \| 1.05 (1.04-1.06, p<0.001) \| 0.96 (0.94-0.97, p<0.001) \| \| MRA \| 1.04 (1.00-1.07, p=0.042) \| 1.07 (1.03-1.11, p<0.001) \| \| Thiazide diuretics \| 1.06 (1.04-1.08, p<0.001) \| 1.01 (0.99-1.03, p=0.191) \| \| Potassium-sparing diuretics \| 0.93 (0.74-1.16, p=0.506) \| 0.95 (0.74-1.20, p=0.659) \| \| Loop diuretics \| 1.44 (1.42-1.46, p<0.001) \| 1.34 (1.32-1.36, p<0.001) \| \| **Cluster (ref: Low-burden)** \|  \|  \| \| Metabolic-vascular \| 2.37 (2.20-2.54, p<0.001) \| 1.87 (1.74-2.01, p<0.001) \| \| Metabolic-vascular * time ^a^ \|  \| p<0.001 \| \| Ischemic \| 1.76 (1.65-1.88, p<0.001) \| 1.24 (1.16-1.33, p<0.001) \| \| Ischemic * time ^b^ \|  \| p<0.001 \| \| Anemia \| 1.92 (1.71-2.15, p<0.001) \| 1.46 (1.30-1.64, p<0.001) \| \| Anemia * time ^c^ \|  \| p=0.258 \| \| Metabolic \| 1.00 (0.92-1.10, p=0.879) \| 1.18 (1.09-1.29, p<0.001) \| \| Metabolic * time interaction ^d^ \|  \| p<0.001 \| \| Abbreviations: HR, hazard ratio; CI, confidence intervals; ref, reference; ACEi, angiotensin-converting-enzyme inhibitors; ARB, angiotensin receptor blocker; MRA, mineralocorticoid receptor antagonists  Coefficients for interaction with time  ^a^ -0.086, p-value<0.001  ^b^ 0.020 , p-value <0.001  ^c^ 0.061, p-value=0.258  ^d^ -0.075, p-value<0.001 \| \| \| |

The interactions between time and cluster were significant for the metabolic-vascular, ischemic and metabolic clusters, denoting the relationship between cluster and death is not constant over time. The interaction between time and the anemic cluster was non-significant. The effect of time on the association between cluster and death was diminished for the metabolic and metabolic-vascular clusters (as denoted by the negative coefficients for the time x cluster interaction from the Cox regression). The opposite was observed for the ischemic cluster, where there was an increase in the risk of death over time, on average.

**Table S13.** Association between mortality and comorbidity cluster with interaction between ejection fraction and cluster (proportional hazards met)

|  | **Univariable HR (95%CI)** | **Multivariable HR (95% CI)** |
| --- | --- | --- |
| Age (years) | 1.06 (1.06-1.06, p<0.001) | 1.05 (1.04-1.05, p<0.001) |
| Sex (Male vs. Female) | 0.98 (0.97-1.00, p=0.009) | 1.07 (0.99-1.15, p=0.087) |
| **Race (ref: White)** |  |  |
| Black | 0.89 (0.87-.091, p<0.001) | 0.90 (0.80-1.01. p=0.057) |
| Hispanic | 0.72 (0.70-0.75, p<0.001) | 0.82 (0.65-1.03, 0.081) |
| Asian | 0.79 (0.75-0.83, p<0.001) | 0.64 (0.44-0.95, p<0.05) |
| Dementia | 2.71 (2.66-2.77, p<0.001) | 1.78 (1.58-1.99, p<0.001) |
| Peptic ulcer | 1.34 (1.30-1.38, p<0.001) | 1.24 (1.10-1.40, p=0.001) |
| Alcohol misuse disorder | 1.11 (1.07-1.15, p<0.001) | 1.38 (1.15-1.64, p<0.001) |
| Hypertension | 2.09 (2.01-2.19, p<0.001) | 1.32 (1.03-1.70, p=0.031) |
| **Education (ref: Bachelor Degree Plus)** | - | - |
| High School Diploma | 1.09 (1.06-1.11, p<0.001) | 1.12 (0.99-1.25, p=0.061) |
| Less than 12 grade | 0.88 (0.77-1.02, p=0.085) | 0.81 (0.11-5.78, p=0.833) |
| Less than Bachelor Degree | 1.03 (1.01-1.06, p=0.003) | 1.01 (0.91-1.13, p=0.788) |
| Business line (commercial vs: Medicare Advantage) | 0.45 (0.44-0.46, p<0.001) | 0.89 (0.80-0.99, p=0.036) |
| Place of diagnosis (inpatient vs. outpatient | 1.19 (1.17-1.21, p<0.001) | 1.27 (1.18-1.37, p<0.001) |
| **Medications at baseline** |  |  |
| Cardioselective beta-blockers | 1.14 (1.13-1.16, p<0.001) | 0.93 (0.86-1.00, p=0.041) |
| Noncardio-selective beta-blockers | 0.94 (0.92-0.96, p<0.001) | 1.03 (0.93-1.14, p=0.543) |
| ACEIs/ARBs | 1.05 (1.04-1.06, p<0.001) | 0.88 (0.82-0.95, p=0.001) |
| MRA | 1.04 (1.00-1.07, p=0.042) | 0.96 (0.81-1.13, p=0.616) |
| Thiazide diuretics | 1.06 (1.04-1.08, p<0.001) | 1.01 (0.92-1.11, p=0.857) |
| Potassium-sparing diuretics | 0.93 (0.74-1.16, p=0.506) | 1.53 (0.49-4.77, p=0.460) |
| Loop diuretics | 1.44 (1.42-1.46, p<0.001) | 1.39 (1.29-1.50, p<0.001) |
| **Cluster (ref: Low-burden)** | - | - |
| Metabolic-vascular | 1.71 (1.68-1.75, p<0.001) | 1.56 (1.36-1.78, p<0.001) |
| Ischemic | 2.06 (2.02-2.10, p<0.001) | 1.52 (1.34-1.73, p<0.001) |
| Anemia | 1.75 (1.69-1.81, p<0.001) | 1.80 (1.43-2.25, p<0.001) |
| Metabolic | 0.78 (0.76-0.80, p<0.001) | 1.02 (0.86-1.21, p=0.825) |
| LVEF | 1.001 (1.001-1.002, p<0.01) | 0.99 (0.99-1.00, p=0.594) |
| **Smoking status (ref: Current smoker)** | - | - |
| Never smoked | 0.89 (0.84-0.94, p<0.001) | 0.72 (0.65-0.80, p<0.001) |
| Not currently smoking | 0.87 (0.81-0.93, p<0.001) | 0.73 (0.65-0.82, p<0.001) |
| Previously smoked | 1.08 (1.02-1.14, p=0.008) | 0.86 (0.77-0.95, p=0.003) |
| *Interaction terms* |  |  |
| Anemia * LVEF | 1.01 (1.00-1.03, p=0.04) | 1.01 (1.00-1.03, p=0.142) |
| Ischemic * LVEF | 0.99 (0.99 -1.00, p=0.945) | 1.00 (0.99-1.01, p=0.692) |
| Metabolic-vascular * LVEF | 1.00 (0.99-1.00, p=0.967) | 1.00 (0.99-1.01, p=0.559) |
| Metabolic * LVEF | 1.00 (1.00 – 1.02, p=0.01) | 1.01 (1.00-1.02, p=0.138) |
| Abbreviations: HR, hazard ratio; CI, confidence interval; ref, reference; ACEis, angiotensin-converting-enzyme inhibitors; ARB, angiotensin receptor blockers; MRA, mineralocorticoid receptor antagonists; LVEF, left ventricular ejection fraction | | |

An interaction term was added between EF (as a continuous variable) and cluster, to study the association with all-cause mortality. There were no significant interactions.

**Table S14.** Association between mortality and comorbidity cluster with interaction between ejection fraction and cluster, including coefficients and standard errors for interaction terms

|  | **Univariable model** | | | **Adjusted model^a^** | | |
| --- | --- | --- | --- | --- | --- | --- |
| **Term** | **Coefficient** | **SE** | ***P* Value** | **Coefficient** | **SE** | ***P* Value** |
| LVEF | 0.00066 | 0.0007 | 0.379 | -0.0012 | 0.002 | 0.613 |
| Anemia | -0.0932 | 0.3812 | =0.806 | -0.1456 | 0.423 | 0.730 |
| Ischemic | 0.7996 | 0.1082 | <0.001 | 0.541 | 0.164 | <0.01 |
| Metabolic-vascular | 0.6699 | 0.1414 | <0.001 | 0.573 | 0.189 | <0.01 |
| Metabolic | -0.6898 | 0.2050 | <0.001 | -0.433 | 0.252 | 0.08 |
| *Interactions* |  |  |  |  |  |  |
| **Anemia * LVEF** | 0.01366 | 0.0064 | 0.035 | 0.011 | 0.007 | 0.132 |
| **Ischemic * LVEF** | -0.00012 | 0.0018 | 0.945 | -0.001 | 0.003 | 0.709 |
| **Metabolic-vascular * LVEF** | 0.0001 | 0.0024 | 0.967 | -0.0018 | 0.003 | 0.606 |
| **Metabolic * LVEF** | 0.00934 | 0.0036 | 0.010 | 0.007 | 0.004 | 0.113 |
| Abbreviations: SE, standard error; LVEF, left ventricular ejection fraction  **^a^** Adjusted for age, sex, race, education, medical insurance status, whether diagnosis was gained in-patient or in out-patient, heart failure medications (angiotensin-converting-enzyme inhibitors, angiotensin receptor blockers, mineralocorticoid receptor antagonists) and smoking status | | | | | | |

**Table S15.** Costs associated with healthcare resource use, per comorbidity cluster, within one-year follow-up (currency United States dollars $)

| **Cost (median, IQR)** | **Low burden** | **Metabolic-vascular** | **Ischemic** | **Anemia** | **Metabolic** | **Overall** | ***P* Value^a^** |
| --- | --- | --- | --- | --- | --- | --- | --- |
| *N* | *24753* | *37011* | *42484* | *6459* | *21208* | *131915* |  |
| Inpatient admissions | 10400 [3350, 25700] | 15600 [6710, 37200] | 12800 [4910, 31100] | 12800 [4730, 30400] | 12800 [5360, 28800] | 13200 [5250, 31600] | <0.001 |
| *N* | *30382* | *45134* | *47694* | *7289* | *27836* | *158335* |  |
| ER visits | 821 [299, 1950] | 1030 [369, 2540] | 798 [267, 1950] | 915 [335, 2200] | 1040 [396, 2520] | 912 [332, 2240] | <0.001 |
| *N* | *66025* | *64773* | *73067* | *12742* | *52529* | *269136* |  |
| Outpatient visits | 1710 [441, 5070] | 3080 [934, 8160] | 2270 [625, 6250] | 2770 [735, 9180] | 2290 [637, 6430] | 2320 [632, 6520] | <0.001 |
| *N* | *78958* | *69040* | *78523* | *13560* | *60222)* | *300303* |  |
| Office visits | 1310 [633, 2440] | 1720 [808, 3210] | 1650 [781, 3090] | 1490 [651, 3170] | 1460 [711, 2740] | 1520 [720, 2880] | <0.001 |
| *N* | *6123* | *13109* | *16323* | *2293* | *4618* | *42466* |  |
| Long-term stays | 423 [191, 894] | 589 [251, 1350] | 531 [229, 1180] | 499 [198, 1100] | 495 [226, 1090] | 523 [227, 1170] | <0.001^b^ |
| *N* | *72729* | *82259* | *82460* | *14753* | *62725* | *314926* |  |
| All medical claims | 5580 [2040, 17200] | 15100 [4940, 41400] | 12700 [4390, 35500] | 11400 [3710, 36500] | 7870 [2780, 22700] | 9700 [3230, 29100] |  |
| *N* | *72729* | *82259* | *82460* | *14753* | *62725* | *314926* |  |
| Overall cost | 7150 [2750, 19300] | 16900 [6000, 43200] | 14400 [5670, 36800] | 13200 [4340, 40000] | 9940 [3630, 25800] | 11500 [4160, 31200] | <0.001 |
| Abbreviations, IQR, inter-quartile range; ER, emergency room  ^a^ Bonferroni adjusted Kruskall-Wallis test was used to compare proportions between clusters (and Dunn post-hoc test to identify significant paired comparisons);  ^b^ Low-burden vs. metabolic not significant | | | | | | | |

**Table S16.** Competing risk analysis (death as competing risk to hospitalization)

|  | **Multivariable HR (95%CI)** |
| --- | --- |
| Age (years) | 0.99 (0.99 – 0.99) |
| Sex (male vs. female) | 0.98 (0.97 – 0.99) |
| **Race (ref: White)** |  |
| Black | 1 (0.99 – 1.02) |
| Hispanic | 0.90 (0.89 - 93) |
| Asian | 0.86 (0.82 – 0.90) |
| Hypertension | 1.58 (1.51 – 1.64) |
| Peptic ulcer | 1.42 (1.40 – 1.46) |
| Dementia | 1.15 (1.13 – 1.77) |
| Alcohol misuse disorder | 1.22 (1.18 – 1.27) |
| **Education (ref: Bachelor Degree Plus)** |  |
| High School Diploma | 1.04 (1.02 – 1.06) |
| Less than 12 grade | 1.04 (0.94 – 1.15) |
| Less than Bachelor Degree | 1.01 (0.99 – 1.03) |
| Commercial vs. Medicare Advantage | 0.88 (0.86 – 0.89) |
| Inpatient vs. outpatient diagnosis | 1.26 (1.25 – 1.27) |
| **Medications at baseline** |  |
| Cardioselective beta-blockers | 0.97 (0.96 – 0.98) |
| Nonselective beta-blockers | 0.99 (0.98 – 1.01) |
| ACEIs/ARBs | 0.98 (0.97 – 0.99) |
| MRA | 0.98 (0.96 – 1.01) |
| Thiazide diuretics | 1.03 (1.01 – 1.05) |
| Potassium-sparing diuretics | 1.22 (1.03 – 1.44) |
| Loop diuretics | 1.17 (1.16 – 1.19) |
| **Comorbidity cluster (ref: Low-burden)** |  |
| Metabolic-vascular | 2.21 (2.17 - 2.25) |
| Ischemic | 2.07 (2.04 – 2.11) |
| Anemia | 1.5 (1.44 – 1.54) |
| Metabolic | 1.16 (1.14 – 1.19) |
| Abbreviations: HR, hazard ratio; CI, confidence intervals; ref, reference; ACEi: angiotensin-converting-enzyme inhibitors; ARB, angiotensin receptor blocker, MRA, mineralocorticoid receptor antagonist | |

An analysis to assess the competing risk of death before admission, was performed with a Fine and Gray model. We estimated cumulative incidence curves. Differences between curves were estimated using Gray’s test for equality, using R package ‘cmprsk’. In the presence of competing risk of death, cluster membership showed an unchanged association with admission to hospital one-year after initial HF diagnosis.
